# *Campylobacter* group II phage CP21 is the prototype of a new subgroup revealing a distinct modular genome organization and host specificity

**DOI:** 10.1186/s12864-015-1837-1

**Published:** 2015-08-22

**Authors:** Claudia Jäckel, Jens A. Hammerl, Jochen Reetz, Andrew M. Kropinski, Stefan Hertwig

**Affiliations:** ᅟ, Federal Institute for Risk Assessment, Department of Biological Safety, Berlin, Germany; Departments of Food Science & Molecular and Cellular Biology & Pathobiology, University of Guelph, Ontario, Canada; Abteilung Biologische Sicherheit, Bundesinstitut für Risikobewertung, Diagnostik und Erregercharakterisierung, Diedersdorfer Weg 1, D-12277 Berlin, Germany

**Keywords:** *Campylobacter*, Phage, Genome, Stability, Repeats

## Abstract

**Background:**

The application of phages is a promising tool to reduce the number of *Campylobacter* along the food chain. Besides the efficacy against a broad range of strains, phages have to be safe in terms of their genomes. Thus far, no genes with pathogenic potential (e.g., genes encoding virulence factors) have been detected in *Campylobacter* phages. However, preliminary studies suggested that the genomes of group II phages may be diverse and prone to genomic rearrangements.

**Results:**

We determined and analysed the genomic sequence (182,761 bp) of group II phage CP21 that is closely related to the already characterized group II phages CP220 and CPt10. The genomes of these phages are comprised of four modules separated by very similar repeat regions, some of which harbouring open reading frames (ORFs). Though, the arrangement of the modules and the location of some ORFs on the genomes are different in CP21 and in CP220/CPt10. In this work, a PCR system was established to study the modular genome organization of other group II phages demonstrating that they belong to different subgroups of the CP220-like virus genus, the prototypes of which are CP21 and CP220. The subgroups revealed different restriction patterns and, interestingly enough, also distinct host specificities, tail fiber proteins and tRNA genes. We additionally analysed the genome of group II phage vB_CcoM-IBB_35 (IBB_35) for which to date only five individual contigs could be determined. We show that the contigs represent modules linked by long repeat regions enclosing some yet not identified ORFs (e.g., for a head completion protein). The data suggest that IBB_35 is a member of the CP220 subgroup.

**Conclusion:**

*Campylobacter* group II phages are diverse regarding their genome organization. Since all hitherto characterized group II phages contain numerous genes for transposases and homing endonucleases as well as similar repeat regions, it cannot be excluded that these phages are genetically unstable. To answer this question, further experiments and sequencing of more group II phages should be performed.

**Electronic supplementary material:**

The online version of this article (doi:10.1186/s12864-015-1837-1) contains supplementary material, which is available to authorized users.

## Background

*Campylobacter* is one of the most important foodborne pathogens worldwide. The genus comprises many species of which the thermophilic *C. jejuni* and its close relative *C. coli* are reported as the most common causes of acute bacterial enteritis [1]. The bacteria are commensals of the gastrointestinal tract of various mammals and birds and are frequently found in chicken flocks where they may spread rapidly [2]. Human infections are caused mainly by the consumption of undercooked meats, especially poultry [3]. To reduce the number of *Campylobacter* along the food chain, various biosecurity measures and post-slaughter decontamination procedures have been tested. As these measures are expensive and not always efficient and since suitable vaccines are not available, phages might be an appropriate means to reduce *Campylobacter* counts in chicken and on chicken products [4]. Indeed phage administration in the laboratory reduced *C. jejuni* colonization of the broiler gut and the contamination on chicken skin by several orders of magnitude [1, 4–16]. A phage cocktail applied via drinking water also efficiently reduced *Campylobacter* counts in chicken on a commercial broiler farm [15]. Phages intended for therapeutic applications or for the control of pathogens in food production have to fulfil a number of requirements to avoid any undesired side effect. Amongst others, the phage genomes have to be stable and free from critical genes (e.g., toxin genes) [17, 18]. Therefore, a basic understanding of the biology and genetics of the phages to be used is of great importance [19]. According to their genome size, *Campylobacter* phages described so far are classified into three groups. While group I phages (~320 kb) have been rarely isolated and have not been used for applications as yet, members of group II (~185 kb) and group III (~135 kb) are very common [20]. Group II phages might be best suited for the reduction of *Campylobacter* since they can infect both *C. jejuni* and *C. coli* strains. Though, the successive application of a group III and a group II phage reduced the numbers of *C. jejuni* in chickens most efficiently [21]. Up to now three (CP220, CPt10, CP21) group II phages have been completely sequenced while vB_CcoM-IBB_35 (IBB_35) is represented, in GenBank, in the form of five contigs [11, 22, 23]. The phages revealed strong DNA homologies to each other whereas only little DNA sequence similarities have been detected to group III phages [24]. Group II *Campylobacter* phages contain e.g., genes for membrane proteins, transposases, and metabolic enzymes including up to twelve proteins with *S*-adenosylmethionine (SAM) domains that are absent in group III [11]. Conversely, group III phages show an expansion of homing endonucleases, represented by up to nine ORFs, that are much less frequently occurring in group II. However, at the protein level both groups showed some relationship to T4-type phages [25]. A striking feature of CP220, CPt10 and CP21 is that their genomes are composed of large modules separated by long DNA repeat regions, which could lead to rearrangements [11, 23]. While the genomes of CP220 and CPt10 are collinear, the genome of CP21 exhibits considerable rearrangement of homologous modules [19, 24]. Functional modules are common in temperate and virulent phages [26]. They are shuffled by recombination which may result in new combinations of modules and the emergence of novel phages [27]. The frequency of recombination events largely depends on the phylogenetic distance between the respective hosts [28]. Illegitimate recombination events are rather rare and may often lead to defective particles but can take place at nearly random points across the genome. On the other hand, homologous recombinations of modules at specific linker sequences occur more frequently, particularly in closely related phages like those belonging to the lambdoid phage family [29, 30].

In this work, the genome organization of CP21 was analysed in detail with special focus on the repeat regions which impede the elucidation of the genome structure of group II phages. On the basis of the available group II sequences, a PCR typing system was established to study the modular genome arrangement of other yet not characterized group II phages. The system was also used to unravel the genome organization of phage IBB_35. We show that group II phages can be allocated to at least two subgroups which revealed distinct host ranges.

## Methods

### Bacterial strains and culture conditions

Most strains originated from the Campynet (CNET) strain collection hosted by the Leibniz Institute DSMZ-German Collection of Microorganisms and Cell Cultures, Braunschweig, Germany, (search term “campynet”). The remaining strains were taken from the *Campylobacter* strain collection of the National Reference Laboratory for *Campylobacter* at the Federal Institute for Risk Assessment (Bundesinstitut für Risikobewertung (BfR)). If not otherwise indicated, the *C. coli* strain NCTC12668 (National Collection of Type Cultures, NCTC, Health Protection Agency, UK) was used for phage propagation and plaque assays [20]. The cultivation of bacteria was performed as previously described [31].

### Isolation, propagation and purification of group II *Campylobacter* phages

The phages CP7, CP21, and CP68 were isolated from poultry produced on organic farms in Berlin, Germany. To recover phages, samples were suspended in SM-buffer and incubated overnight at 45 °C on a stirrer. Thereafter, the samples were centrifuged at 10,000 × g followed by filtration of the supernatants through 0.22 μm nitrocellulose membrane filters (VWR International, Darmstadt, Germany). Phage activity was determined by spot tests on lawns of the reference strains *C. jejuni* NCTC11168 and *C. coli* NCTC12668 [20]. Three previously described [20] group II phages (NCTC12675, NCTC12683 and NCTC12684 termed CP75, CP83 and CP84, respectively), obtained from NCTC were included in the study. All phages were purified by three consecutive single plaque-assays. High-titre lysates of the phages were obtained by infecting 1 l cultures of the indicator strain (OD_588_ of ~0.4) with phages at a MOI of ~0.01 for 24 h. To remove remaining cells and debris, lysates were centrifuged for 30 min at 10,000 × g and then filtrated (see above). Phages were concentrated by ultracentrifugation and purified by CsCl step gradients [32]. Plaque assays, single plaque isolations, and propagation of the phages were performed as previously described [31].

### Host range determination

The host range of the phages was determined by activity assays. 400 μl of the respective indicator strain were mixed with 6 ml prewarmed NZCYM soft agar (0.6 %) and poured onto a LB agar plate. Ten μl of each lysate (adjusted to ~5 × 10^7^ pfu/ml) were spotted onto the overlay agar. Plates were incubated overnight at 42 °C under microaerobic conditions [31].

### Transmission electron microscopy (TEM)

CsCl-purified phages were investigated by TEM using the negative staining procedure with ammonium molybdate. Briefly, drops of phage preparations were applied to pioloform-carbon-coated, 400-mesh copper grids (Plano GmbH, Wetzlar, Germany), incubated for 10 min and fixed with 2.5 % aqueous glutaraldehyde (Taap Laboratories, Aldermaston, United Kingdom) for 1 min. Thereafter, phages were stained with a 3 % aqueous ammonium molybdate (Merck, Darmstadt, Germany) solution (pH 7.0) for 3 min. Specimens were examined by TEM using a JEM-1010 (JEOL, Tokyo, Japan) at 80 kV accelerated voltage.

### Mass spectrometrical analysis of structural proteins

For the determination of CP21 structural proteins, CsCl-purified phages were disintegrated by boiling in SDS buffer for 10 min. Proteins were subjected to SDS-PAGE and separated at 20 mA on a one-dimensional 15 % (wt/vol) gel at 15 °C [33]. Bands of interest were excised and prepared for tryptic *in gel*-digests as previously described. The prepared samples were subjected to tandem matrix-assisted laser desorption ionization-time-of-flight mass spectrometry (MALDI-TOF-TOF MS/MS) analysis. Mass spectra were analyzed and interpreted using the Mascot software (Matrix Science Ltd., London, United Kingdom) followed by NCBI nr database (NCBI) searches.

### Isolation of phage DNA, restriction analysis and PCR

Isolation of phage DNA was conducted by incubating purified phage particles (400 μl) in 10 mM Tris–HCl (pH 7.5), 1 mM EDTA, 0.5 % SDS supplemented with 2 μl of Proteinase K (20 mg/ml) at 56 °C for 2 h followed by ethanol precipitation. To analyse restriction patterns, phage DNAs were digested with restriction endonucleases (Fermentas, St. Leon-Roth, Germany) according to the manufacturer’s recommendations. Restriction fragments were separated by agarose gel electrophoresis. In general, PCR analyses were performed in an Eppendorf Mastercycler ep Gradient (Eppendorf, Hamburg, Germany) according to standard protocols as previously described [32]. Single reactions were carried out in a final volume of 50 μl by the use of the DreamTaq DNA polymerase amplification components (Fisher Scientific, Schwerte, Germany). The mastermix of a reaction comprised 26.75 μl RNase-free water, 5.0 μl 10 × DreamTaq Buffer, 5 μl dNTP solution (2 mM), 1.25 μl of DreamTaq Enzyme, 5 μl of each primer and 2.0 μl of template DNA (10–20 ng/μl). Due to the low G + C content of *Campylobacter* phage genomes, conditions (annealing temperature and elongation time) of the thermal/time profile of the PCR protocol had to be adapted as stated below. For the determination of the modular structure of the *Campylobacter* group II phages, the same PCR protocol was used: initial PCR activation and template denaturation at 94 °C for 120 s followed by 35 cycles including denaturation phase at 94 °C for 15 s, annealing at 47 °C for 15 s and elongation for 210 s at 72 °C. In addition, the protocol contained a final elongation step at 72 °C for 1 min before the PCR reactions were stored at 4 °C until further processing.

### Sequence determination and bioinformatic analysis of the CP21 genome

Whole-genome sequencing was performed by LGC Genomics (Berlin, Germany) as previously described [23]. Library generation for 454 FLX sequencing was carried out according to the manufacturer’s standard protocols (Roche/454 Life Sciences, Branford, Connecticut, USA). Phage DNA was sheared by nebulization into fragments ranging in size from 500 to 1000 bp. The fragments were end-polished and the 454 A and B adaptors required for the emulsion PCR and sequencing were added to the ends of the fragments by ligation. The concentration of the resulting fragment library was measured by fluorometry (Qubit 2.0, Life Technologies, Darmstadt, Germany) and sequencing was performed on 1/16 picotiterplate (PTP) on the GS FLX using Roche/454 Titanium chemistry. A total of 55,386 sequence reads were assembled using the Roche/454 Newbler software at default settings (454 Life Sciences Corporation, Software release 2.3 (091027_1459)). Assembly resulted in three independent contigs (CP21_C1, 94,267 bp; CP21_C2, 56,052 bp and CP21_C3, 28,698 bp) with an average sequence coverage of more than 105 per consensus base. Gaps were closed by PCR and Sanger sequencing. To determine the sequences of repeat regions (RR), PCR primers were deduced from their flanking sequences. The repeat regions were amplified by PCR and used as targets for *in vitro* transposon mutagenesis using the Epicentre EZ < TNekTET-1 Insertion Kit (Biozym, Hessisch Oldendorf, Germany) according to the manufacturer’s recommendations. Mutagenized PCR products were inserted into pLitmus38 (Ap^r^, New England Biolabs, Frankfurt am Main, Germany) and introduced into *E. coli* strain Genehogs (Invitrogen, Karlsruhe, Germany). After transformation, transformants were selected on agar containing tetracycline (12.5 μg/ml). Nucleotide sequences of eight to twelve transformants with transposon insertions at different positions within each repeat region were used to determine the whole sequences of the repeat regions. Sequencing was performed using primers deduced from the marker gene of the EZ < TNekTET-1 Insertion Kit (Biozym). The initial nucleotide sequence of the CP21 draft genome was submitted to EMBL under the accession number HE815464 [23]. As the 454 sequencing technique is prone to frameshift errors at homopolymer stretches [34], the genome sequence was verified at 70 critical regions. An updated CP21 genome sequence was submitted to GenBank in March 2015. Sequence analyses and alignments were carried out using the Accelrys DS Gene software package of the Accelrys Inc. (USA). Putative open reading frames (ORFs) were suggested using the algorithm of the RAST server [35–37]. Similarity and identity values were calculated using different BLAST algorithms (http://www.ncbi.nlm.nih.gov/BLAST/) at the NCBI homepage [38]. Putative Rho-independent transcription terminators were identified using TransTerm [39] and Arnold (http://rna.igmors.u-psud.fr/toolbox/arnold/).

## Results

### Morphology and host range of CP21

**Fig. 1:**
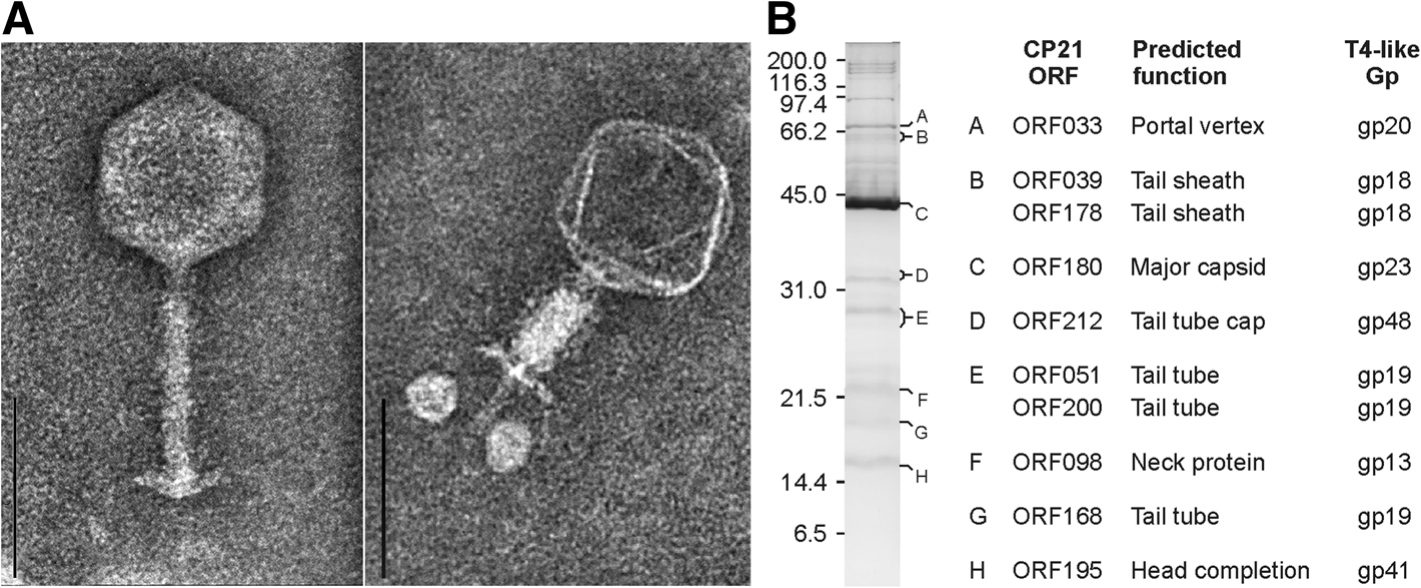
Morphology and structural proteins of CP21. **a** Electron micrographs of CP21 phage particles with a non-contracted and a contracted tail. The bars correspond to 100 nm. **b** SDS-PAGE and mass spectrometric analysis of structural proteins

Phage CP21 showed the typical morphology of a myovirus (Fig. 1a) with an isometric head (diameter = 93 nm) and a long contractile tail (135 × 24 nm). Thus, it fits well into *Campylobacter* phage group II whose members have longer tails than group III phages [24]. As with other *Campylobacter* phages, CP21 has a neck without a collar, and a small base plate. Its tail fibers are fragile and could therefore not be measured. For the determination of the CP21 host range, 255 strains belonging to six *Campylobacter* species were tested by spot assays. Phage activity was limited to the species *C. jejuni* and *C. coli*, of which 13 out of 227 and four out of 18 strains, respectively, were lysed (Table 1) (see also in Additional file 1: Table S1).

**Table 1:** Host ranges of the investigated *Campylobacter* group II phages

*Campylobacter* phage	CP68	CP75	CP84	CP7	CP83	CP21	IBB_35 ^a^
Infected strains	31 (255)	30 (255)	34 (255)	19 (255)	15 (255)	17 (255)	12 (90)^a^
% positive	12.2	11.8	13.3	7.5	5.9	6.7	13.3
*C. jejuni s*trains	21 (227)	20 (227)	24 (227)	14 (227)	13 (227)	13 (227)	5 (76)^a^
% positive	9.3	8.8	10.6	6.2	5.7	5.7	6.6
*C. coli* strains	10 (18)	10 (18)	10 (18)	5 (18)	2 (18)	4 (18)	7 (14)^a^
% positive	55.6	55.6	55.6	27.8	11.1	22.2	50.0
*C. lari* strains	0 (5)	0 (5)	0 (5)	0 (5)	0 (5)	0 (5)	0 (5)
% positive	0	0	0	0	0	0	0
*C. fetus* strains	0 (2)	0 (2)	0 (2)	0 (2)	0 (2)	0 (2)	0 (2)
% positive	0	0	0	0	0	0	0
*C. sputorum* strains	0 (2)	0 (2)	0 (2)	0 (2)	0 (2)	0 (2)	0 (2)
% positive	0	0	0	0	0	0	0
*C. hyointestinalis* strains	0 (1)	0 (1)	0 (1)	0 (1)	0 (1)	0 (1)	0 (1)
% positive	0	0	0	0	0	0	0
Phage subgroup	A	A	A	B	B	B	(A)

^a^For the determination of the IBB_35 host range, only *C. jejuni* and *C. coli* strains were tested that were lysed by any other phage

### Phage CP21 is closely related to other group II phages

The genomic sequence of CP21 was determined by 454 technology [23]. As this technique is prone to frameshift errors at homopolymer stretches [34], more than 70 critical regions of the CP21 genome showing striking deviations (e.g., a stop codon instead of an amino acid codon) to nearly identical sequences of other group II phages were amplified by PCR and resequenced using the Sanger method. After verification, the published CP21 nucleotide sequence [23] has been revised at these positions. Due to the circular permutation of the CP21 genome and to facilitate comparisons with related phages, the end points of the genome were defined in accordance with the CP220 genome [11].

**Fig. 2:**
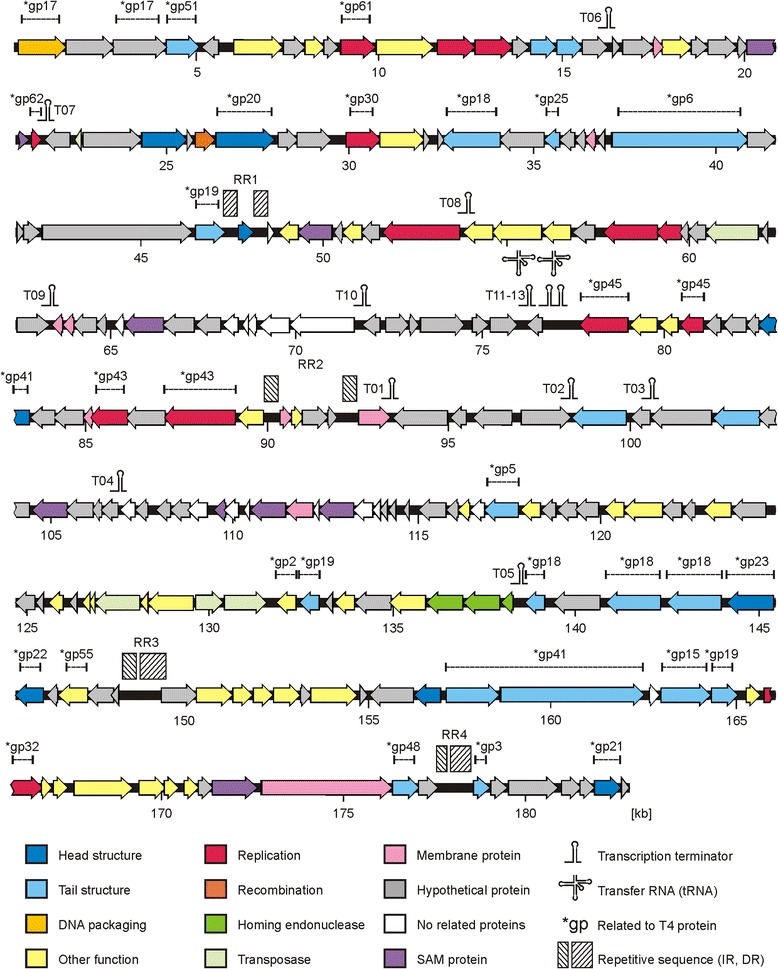
Genetic map of the CP21 genome. Putative genes are coloured according to the predicted functions of their products. The position of putative Rho-independent transcription terminators, tRNA genes and repeat regions are indicated

The CP21 genome (182,761 bp) showed strong overall DNA homologies (BLASTN) to the completely sequenced group II phages CP220 (Total score: 3.745E + 05, Query cover: 82 %, E value: 0.0, Identity: 94 %) and CPt10 (Total score: 3.213E + 05, Query cover: 84 %, E value: 0.0, Identity: 95 %) and also to the five contigs of phage IBB_35 (Total score: 2.311E + 05, Query cover: 85 %, E value: 0.0, Identity: 94 %). However, the CP21 genome is 5.3 kb and 7.0 kb larger than the genomes of CP220 and CPt10, respectively (Table 2). Several DNA regions, 0.4 to 3 kb in size, are missing or relocated in the other group II phages (see in Additional file 1: Table S2). Some of them encode homing endonucleases and transposases that may have caused deletions or genomic rearrangements. This is clearly discernible in the region between the positions 22.3 kb and 22.7 kb which in CP21 harbors a transposase gene flanked by short DNA repeats. While repeats also exist at a similar position in CP220 and CPt10, the transposase gene is located more than 100 kb apart in these two phages. Altogether, CP21 contains eight homing endonuclease and transposase genes whereas only three to five of these genes were detected in the other group II phages (Fig. 2) (see also in Additional file 1: Table S3). Another striking difference between the phages is the fact that unlike CP220, CPt10 and IBB_35 which contain tRNA genes for arginine and tyrosine, CP21 possesses tRNA genes for threonine and proline (Table 2).

**Table 2:** Properties of the hitherto sequenced *Campylobacter* group II phages

	CP21	CP220	CPt10	IBB_35^a^
Family	*Myoviridae*	*Myoviridae*	*Myoviridae*	*Myoviridae*
Head diameter (nm)	93	96	n.a.	100
Tail dimensions (nm)	135 × 24	110 × 16	n.a.	140 × 17
Host species	*C. jejuni, C. coli*	*C. jejuni, C. coli*	*C. jejuni, C. coli*	*C. jejuni, C. coli*
Genome size according to PFGE (kb)	209	197	176	204
Genomic sequence (bp)	182,761	177,493	175,720	172,065
G + C content (%)	27.2	27.4	27.3	27.4
Total no. of ORFs	220	194	201	210
Forward strand	91	173	180	n.a.^b^
Reverse strand	129	21	21	n.a.^b^
Coding density (%)	88.2	88.4	89.7	90.0
Gps with predicted function	78 (35 %)	74 (38 %)	70 (35 %)	84 (40 %)
Transcription terminators	13	11	9	14
tRNAs	2 (Thr, Pro)	2 (Arg, Tyr)	2 (Arg, Tyr)	2 (Arg, Tyr)
Homing endonucleases	3	1	1	3
Transposases	5	4	2	2
T4-related proteins	32	39	34	35
SAM-related proteins	8	11	12	6
Genome structure	Linear	Linear	Linear	n.a.
Sequence state	Complete	Complete	Complete	Incomplete^a^
Accession number	NC019507.1	FN667788	FN667789	C1:HM246720
				C2:HM246721
				C3:HM246722
				C4:HM246723
				C5:HM246724
Reference	[23]	[11]	[11]	[22]

*SAM* S-Adenosyl-L-Methionine, *ORF* Open Reading Frame, *PFGE* Pulsed-Field Gel Electrophoresis, *n.a*. not available, *gps* gene products

^a^The genome characteristics of IBB_35 base on five annotated contigs

^b^According to the suggested arrangement of the five contigs, the majority of ORFs (68 %) are located on the forward strand [22]

**Fig. 3:**
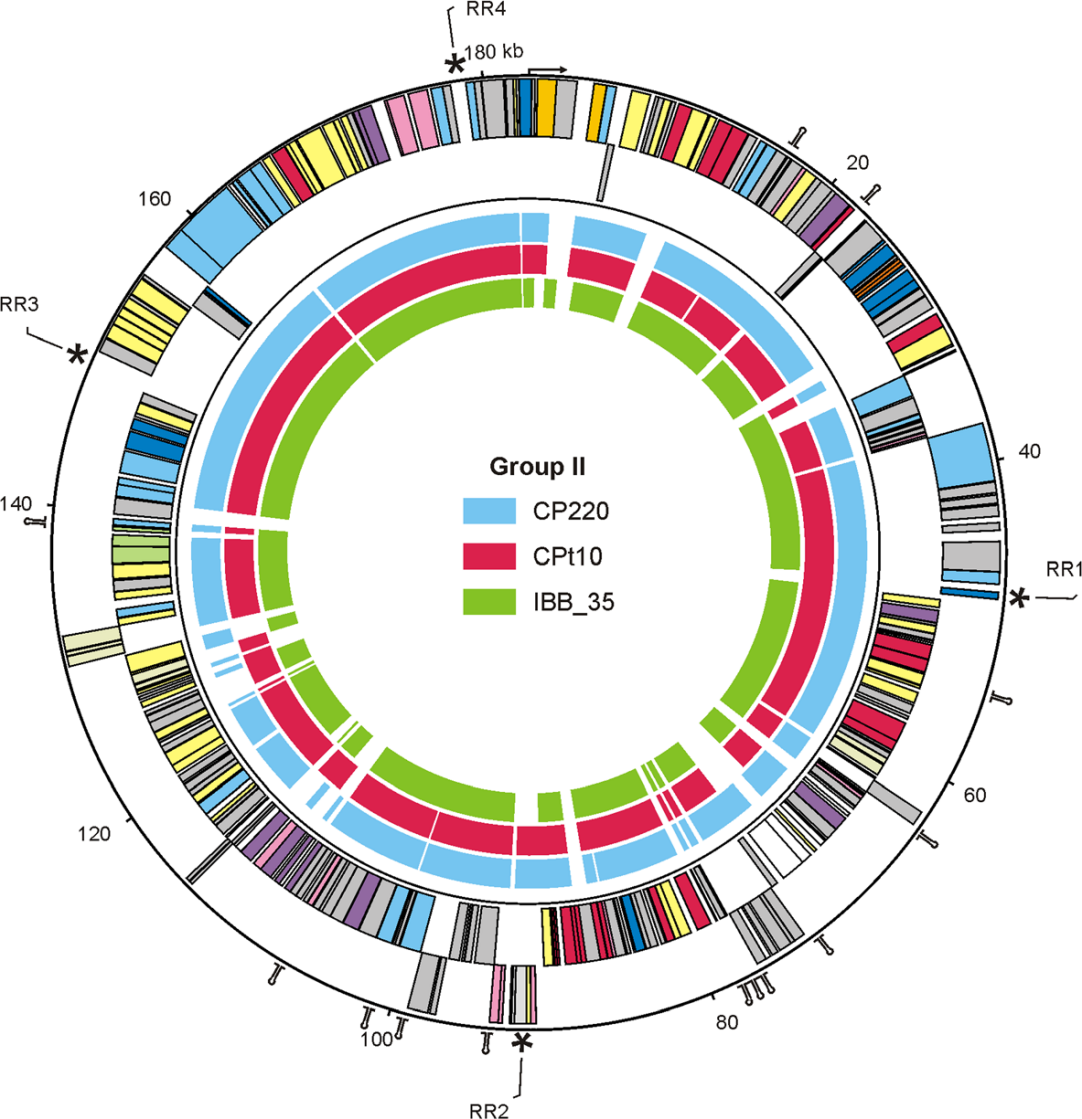
Group II phages contain very similar proteins. Circular illustration (synteny plot) of the CP21 genome and similarities of predicted products to proteins of the *Campylobacter* group II phages CP220, CPt10 and IBB_35. Positions of the CP21 repeat regions and of putative Rho-independent transcription terminators (Ω) are indicated

**Fig. 4:**
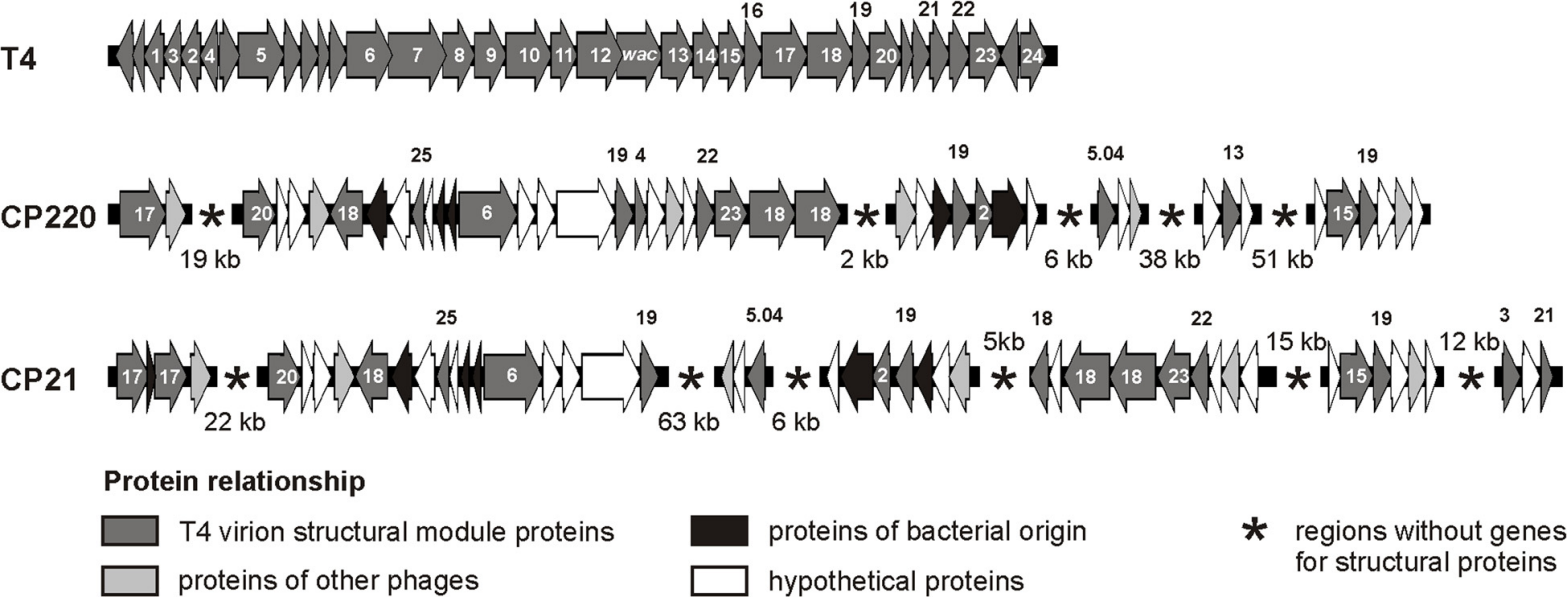
Arrangement of structural genes in T4, CP220 and CP21. Genes are denoted according to the nomenclature of T4 and functionally assigned. *wac*, whisker antigen control gene

At the protein level, 153 out of 220 (69.5 %) predicted CP21 products are more than 90 % identical to CP220 proteins (Fig. 3) (see also in Additional file 1: Table S3). Only for 22 products, no counterparts could be found in CP220, CPt10 and IBB_35. The function of most of these proteins is unknown. CP21 revealed almost no DNA sequence relatedness to group III phages. Though, a number of proteins, most of them involved in phage assembly and replication, show some similarity to products of group III phages. These common proteins of group II and group III phages represent modules (virion structural module, DNA replication module) that are typically found in T4-type phages and make up the core genome. A comparison of virion structural modules (T4 gp1 to gp24) demonstrated the distant relationship of CP21 and CP220 and also of group III phage CP81 to T4-type phages (Fig. 4) [23]. Using mass spectrometry, a number of CP21 structural proteins similar to T4 head and tail proteins have been identified (Fig. 1b).

### CP21 and CP220/CPt10 possess different genome organizations

**Fig. 5:**
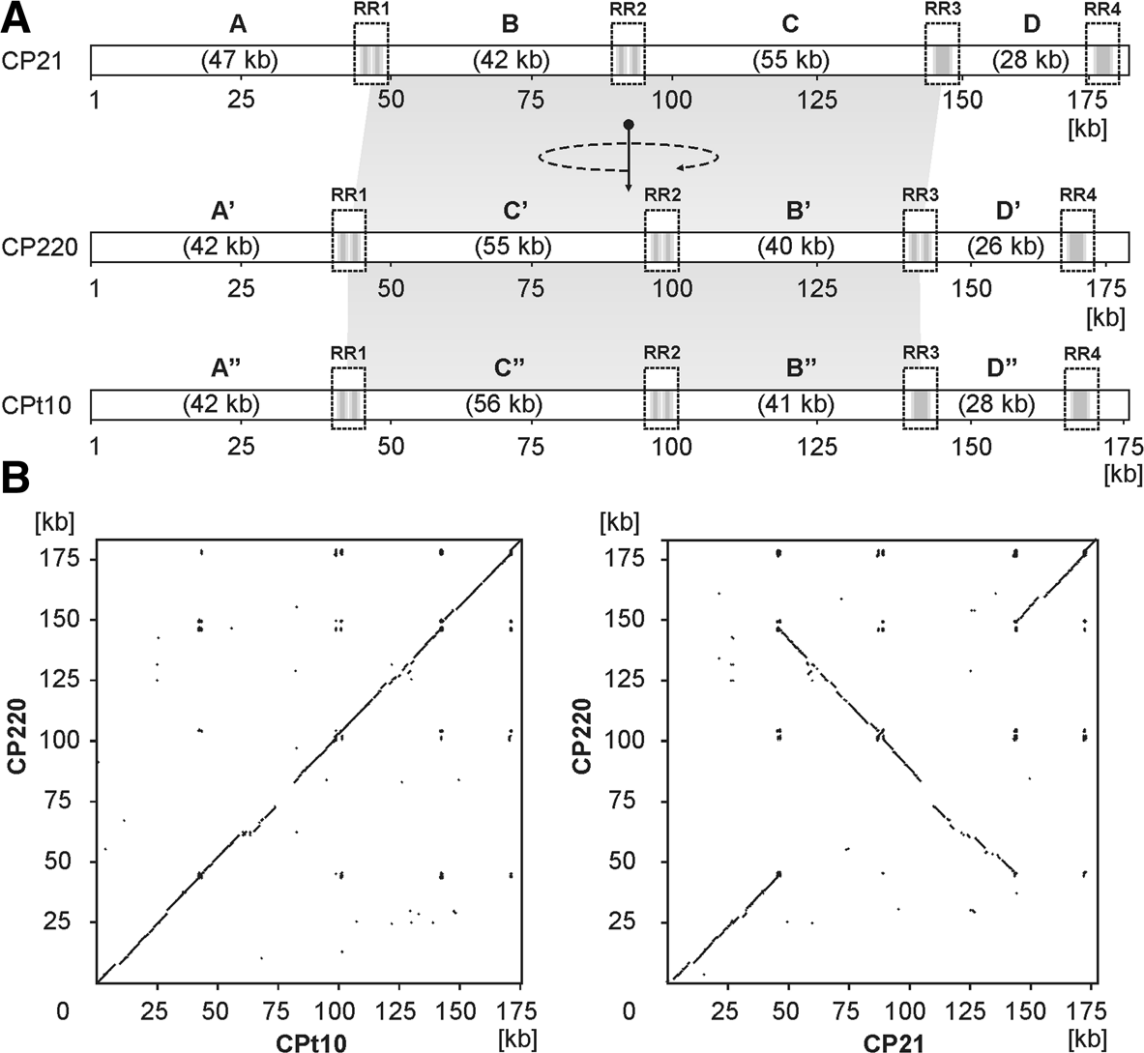
Genome organization and DNA homologies of CP21, CP220 and CPt10. **a** Modular composition of the genomes. The modules A, B, C, and D are separated by long repeat regions (RR). In CP21 module B and *C* are inverted. **b** Dot plot alignment of CP220 with CPt10 and CP21. Alignments were performed with the DS Gene software package of the Accelrys Inc. (USA). The numbers on the axes give the scale for the genomes in kilo bases

While the arrangement and orientation of CP220 structural genes bear some resemblance to the modules of T4-type phages, the location and orientation of the corresponding CP21 genes is quite diverse (Fig. 4). The same holds true for genes involved in phage replication. Both structural and replication genes are not clustered in CP21. Instead, these genes are widely dispersed on the genome (Fig. 2). Similar to CP220 and CPt10, CP21 contains four closely related repeat regions (RR1 to RR4, 1 to 2.9 kb in size) which divide the genome into four modules (A to D, 28 to 55 kb, Fig. 2 and Fig. 5a), three of which carrying genes for structural and replication proteins. With the exception of one repeat region in CP220 and CPt10 [11], the remaining regions are extragenic. They are similarly located on the three phage genomes and show strong DNA homologies to each other. The dot plot alignment of the phages CP21 and CP220/CPt10 showed that their genomes are closely related but differently organized (Fig. 5b). A closer view at the gene maps disclosed that two (B and C) of the four CP21 modules are inverted (Fig. 5a). As a result many structural genes located in these regions are divergently orientated in CP21 and CP220/CPt10 (Fig. 4). By contrast, genes situated on module A and D are transcribed in the same direction in all three phages.

### The repeat regions of group II phages are variations of the same theme

**Fig. 6:**
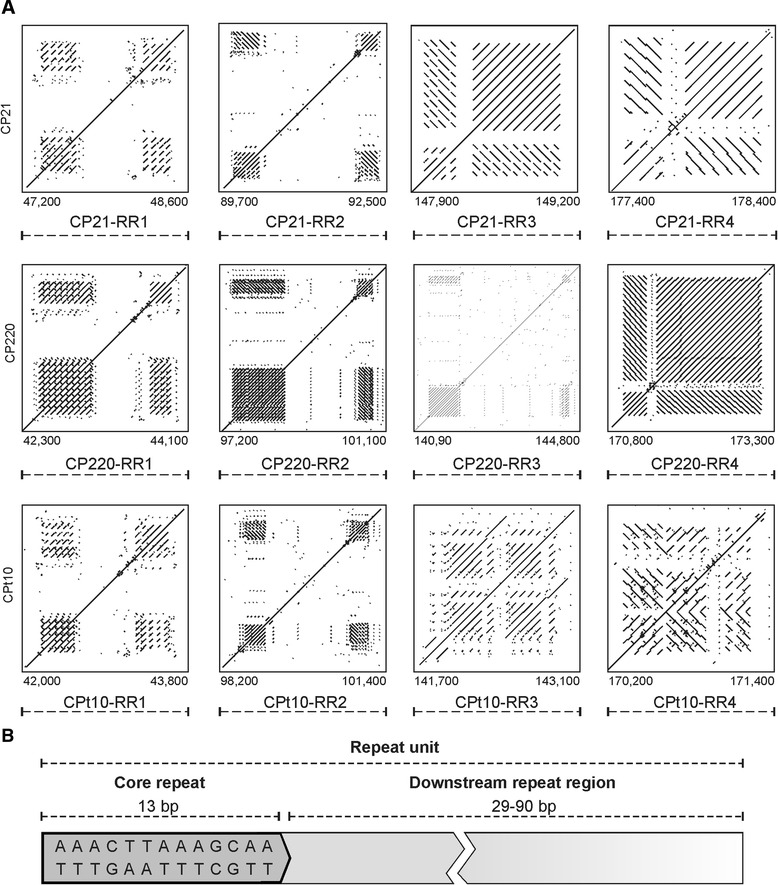
Structure of the repeat regions and repeat units of CP21, CP220 and CPt10. **a** Dot plot analyses of the 12 repeat regions. The positions of the repeat regions on each genome are indicated. **b** Composition of the repeat units

**Fig. 7:**
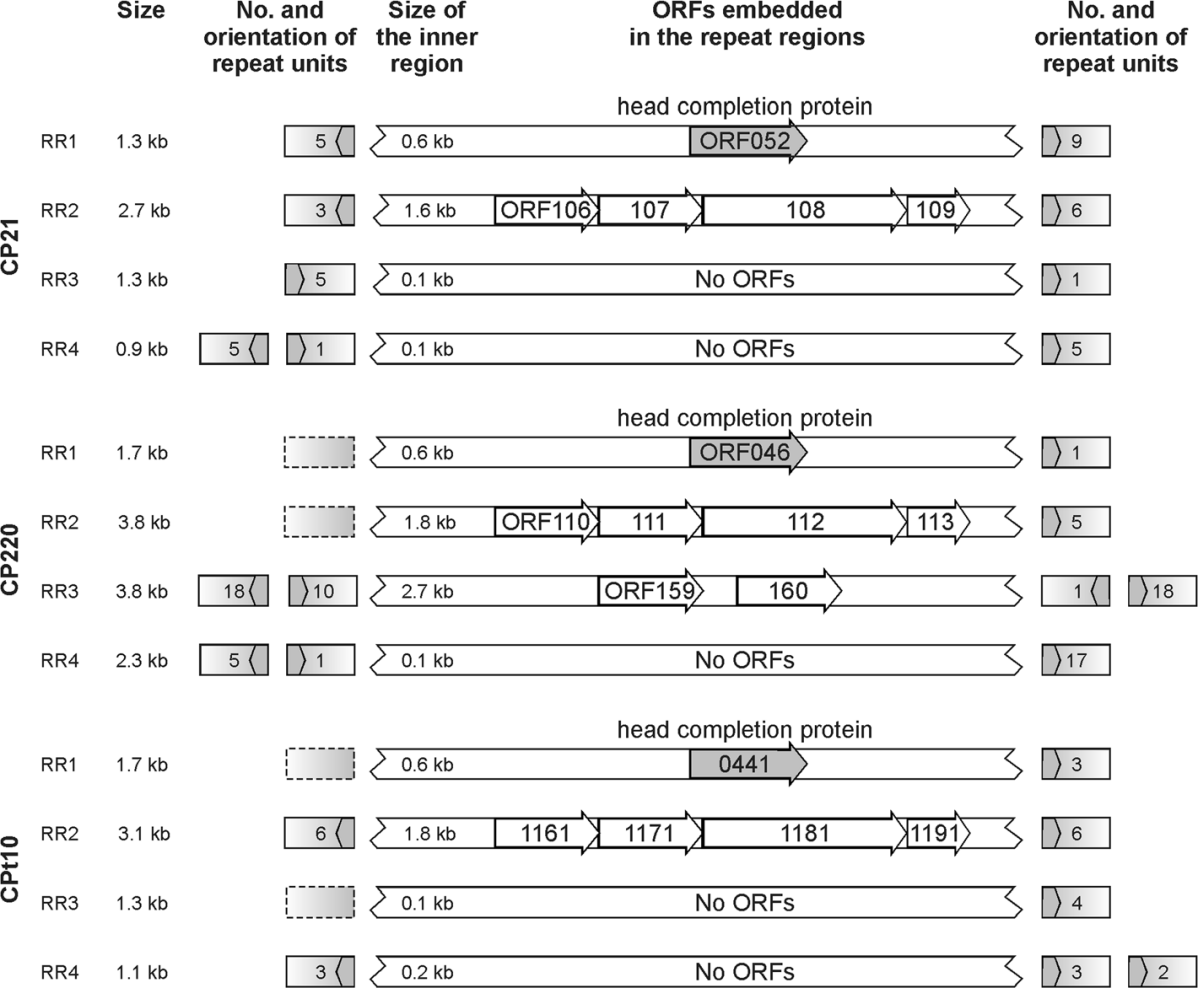
Some repeat regions harbour genes. The size of each repeat region and the number and orientation of the respective repeat units are stated. Empty boxes indicate repeat units that do not contain the complete core sequence. The inner regions represent non-repetitive sequences, some of which encode proteins

The repeat regions RR1 to RR4 of CP21, CP220 and CPt10 have a bipartite structure and are composed of numerous repeat units which encompass stretches of unique DNA sequences (Fig. 6a). Some of the enclosed sequences represent genes, namely ORF052 and the ORFs 106 to 109 within repeat regions RR1 and RR2, respectively (Fig. 7). Notably, the CP220 RR3 repeat region, which is much larger than RR3 of CP21 and CPt10 contains two genes for hypothetical proteins that do not exist in the other two phages. Each arm of a repeat region is composed of up to 18 units, most of them 72 to 86 bp in length (Fig. 6b). A core consensus sequence that was found in all CP21 units (allowing one mismatch) has a length of 13 nucleotides and the sequence 5′-AAACTTAAAGCAA-3′. This core sequence was also identified in most CP220/CPt10 units. The comparison of the repeat regions revealed two peculiarities. Firstly, some regions contain direct, some others inverted repeats (Fig. 6a). While CP21 possesses only one repeat region (RR3) comprising direct repeats, both RR1 and RR3 of CP220/CPt10 contain direct repeats. Thus, RR1 which is located on the left side of the inverted module B has a different structure in CP21 and CP220/CPt10. It is conceivable that this aberration is the result of a recombination event between repeat regions yielding the inversion of the modules B and C. The second striking feature of the repeat regions is the diverse number of units. This finding pertains to both the total number of units and the numbers of units within the two arms of each region, which, in some cases, vary considerably (Fig. 7). It is noteworthy that even the phages CP220 and CPt10 which share the same modular composition contain repeat regions revealing significant discrepancies. This suggests the repeat regions to be targets for recombination. The number of units can of course also be changed via slipped-strand mis-pairing or secondary structure formation during DNA replication [40, 41]. It should be added that the three phages contain additional single units or the core consensus sequence at other positions on their genomes. In CP21 there is e.g., a region (approximate nucleotide position between 27.9 and 29.5 kb) containing several copies of repeat units which, however, show rather extensive sequence polymorphisms (see in Additional file 1: Figure S1A). These sites might be remnants of former recombination or slipped-strand mis-pairing events. CP220 and CPt10 possess a similar repeat region, which exhibits a lower complexity than its counterpart in CP21 (see in Additional file 1: Figure S1B and C).

### A modular composition is common in group II phage genomes

**Fig. 8:**
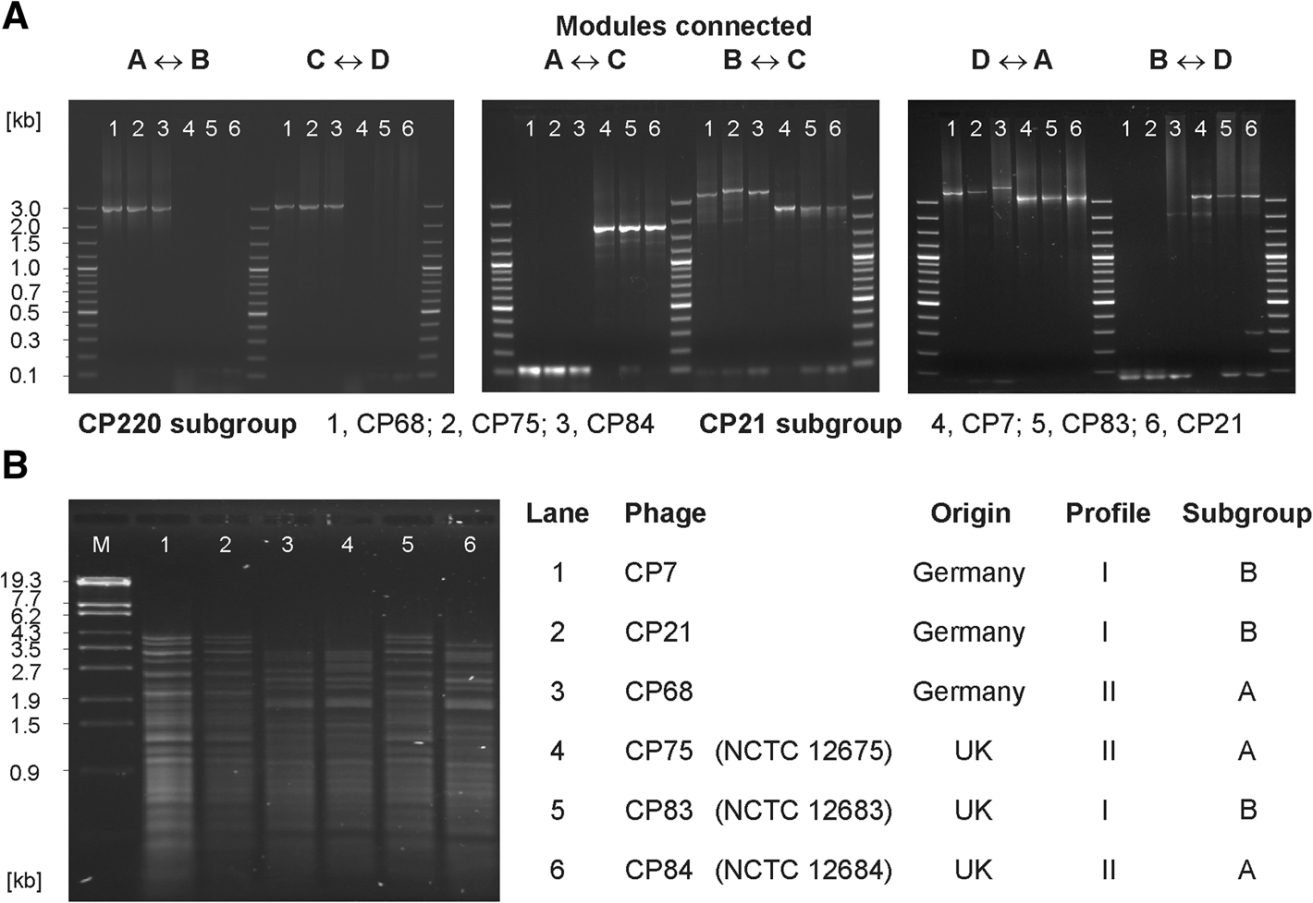
Classification of the investigated phages into two subgroups. **a** PCR analysis of the modular composition of the phage genomes. Lane 1, lane 2, lane 3, lane 4, lane 5, lane 6 show amplicons obtained with the phage DNAs of CP68, CP75, CP84, CP7, CP83, CP21, respectively. **b** VspI restriction patterns of the analysed phages

To examine whether other yet not characterized group II phages also possess a modular genome organization, a PCR system was established that enabled us to determine the order of modules. For this study PCR primers were deduced from conserved sequences flanking the repeat regions of CP21, CP220 and CPt10. Due to the very low GC content of *Campylobacter* phages and since the long repeat regions probably generate secondary structures that might impede their amplification, primers of up to 40 nucleotides in length were designed (see in Additional file 1: Table S4). The primers were used in different combinations to identify several modular compositions. As shown in Fig. 8a, single products similar in size as those of the reference phages were obtained in most PCR reactions. The study disclosed that two phages (CP7, CP83) possess the same modular arrangement like CP21 while three other phages (CP68, CP75, CP84) belong to the subgroup represented by CP220/CPt10. None of the investigated phages showed an individual genome organization. Thus, the hitherto analysed group II phages belong to two subgroups of which CP21 and CP220/CPt10 are the prototypes.

It has already been pointed out that *Campylobacter* phage DNAs can efficiently be cleaved by restriction endonucleases recognizing sheer AT-sequences [25]. We now compared VspI restriction patterns of group II phages belonging to different subgroups. While members of each subgroup exhibited very similar or even identical restriction patterns, the profiles of phages belonging to different subgroups were more divergent (Fig. 8b). By *in silico* analysis of the CP21 and CP220 genomic sequences it became evident that the differences in VspI restriction patterns of these phages were mainly caused by their diverging modular organization. Thus, restriction analysis might be a simple method to classify a group II phage into one of the two currently known subgroups or to detect new subgroups.

### Analysis of phage IBB_35

**Fig. 9:**
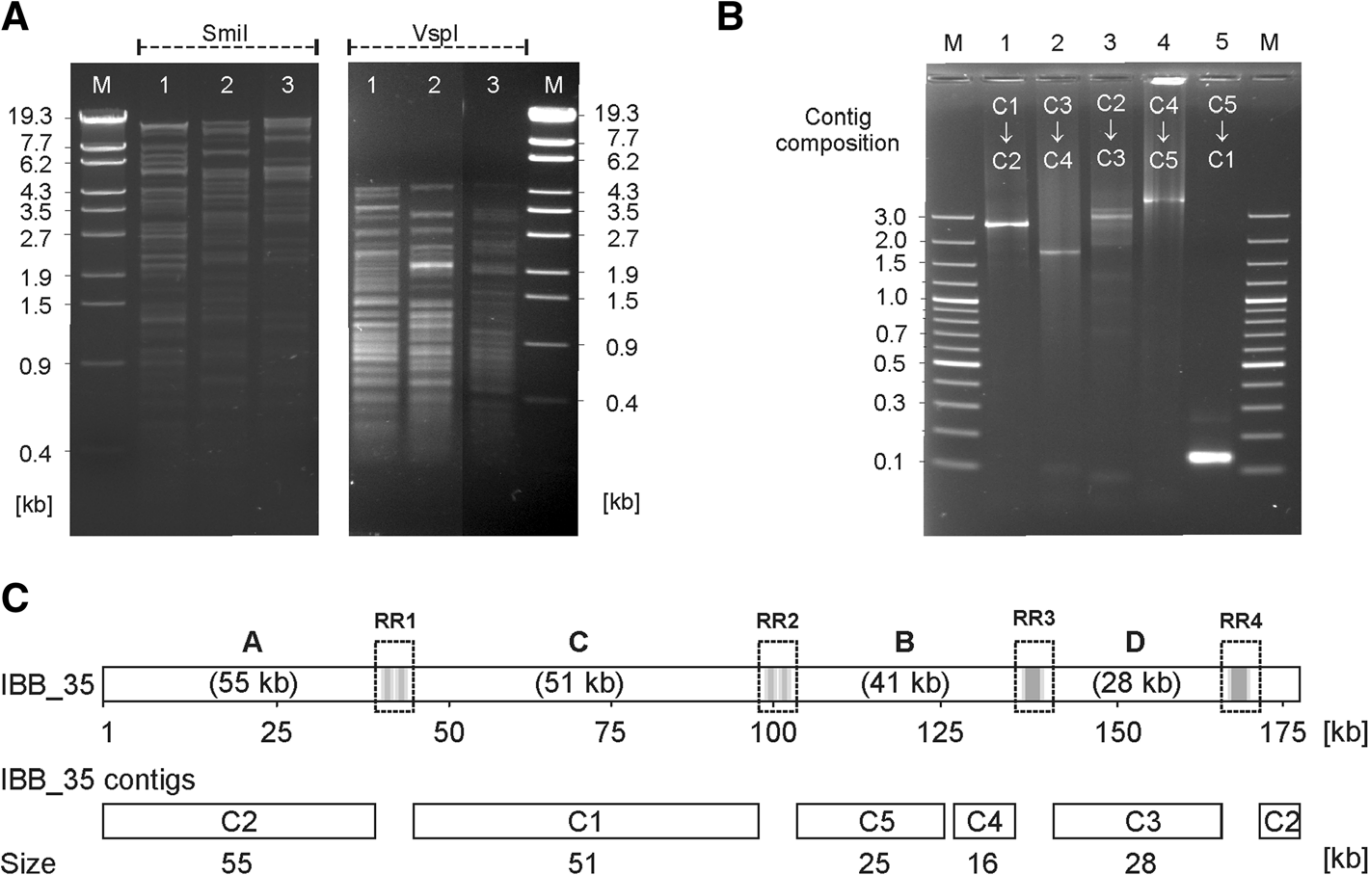
Analysis of the phage IBB_35 genome. **a** SmiI and VspI restriction patterns. Lane 1, CP21, lane 2, IBB_35, lane 3, CP68. **b** Order of the five IBB_35 contigs (C) determined by PCR. **c** IBB_35 shows the same modular genome organization as CP220

The technique used to study the genome organization of group II phages was also applied to IBB_35. With this phage attempts to close gaps between five contigs were unsuccessful probably due to substances or modifications inhibiting Taq and Φ29 polymerases and DNA restriction [22]. In addition, a striking discrepancy has been observed between the genome sizes calculated by sequencing and pulsed-field gel electrophoresis (173 kb versus 204 kb). At first we determined the susceptibility of IBB_35 DNA to restriction endonucleases which exclusively recognize A/T sequences. As shown in Fig. 9a, DNA of this phage was efficiently cleaved by SmiI and VspI like the DNAs of CP68 (CP220 subgroup) and CP21. However, based on its restriction patterns, IBB_35 could not be clearly allocated to one of the subgroups. In the next step, gaps between the IBB_35 contigs were analysed by PCR. Using different combinations of long primers deduced from the ends of the published IBB_35 sequences, we obtained amplicons that disclosed the arrangement of contigs (Fig. 9b) and demonstrated that IBB_35 possesses the same modular composition as CP220 (Fig. 9c). The study also confirmed that contig 4 and contig 5 are linked and showed that the PCR products (1.5 kb to 3.5 kb) were significantly larger than predicted (100 to 400 bp) [22]. Summing up the sizes of the five contigs and the amplicons, which represent the yet not characterized gaps, a genome size of 180 kb was calculated. To get some information about the DNA regions connecting the contigs, we sequenced the PCR products. Even though we failed to determine the complete sequences of all amplicons, partial sequences were obtained. The data clearly showed that the IBB_35 contigs are indeed linked by repetitive sequences. Furthermore, the core consensus sequence identified within the repeat regions of the other group II phages was also found in IBB_35. In addition, some yet not identified genes probably encoding a head completion protein and other hypothetical proteins were detected in the amplified DNA. These genes are homologous to genes found within the repeat regions RR1 and RR2 of CP21 and CP220/CPt10. Thus, IBB_35 possesses some characteristics typical for a group II phage.

### Each phage subgroup exhibited a distinct host range

**Fig. 10:**
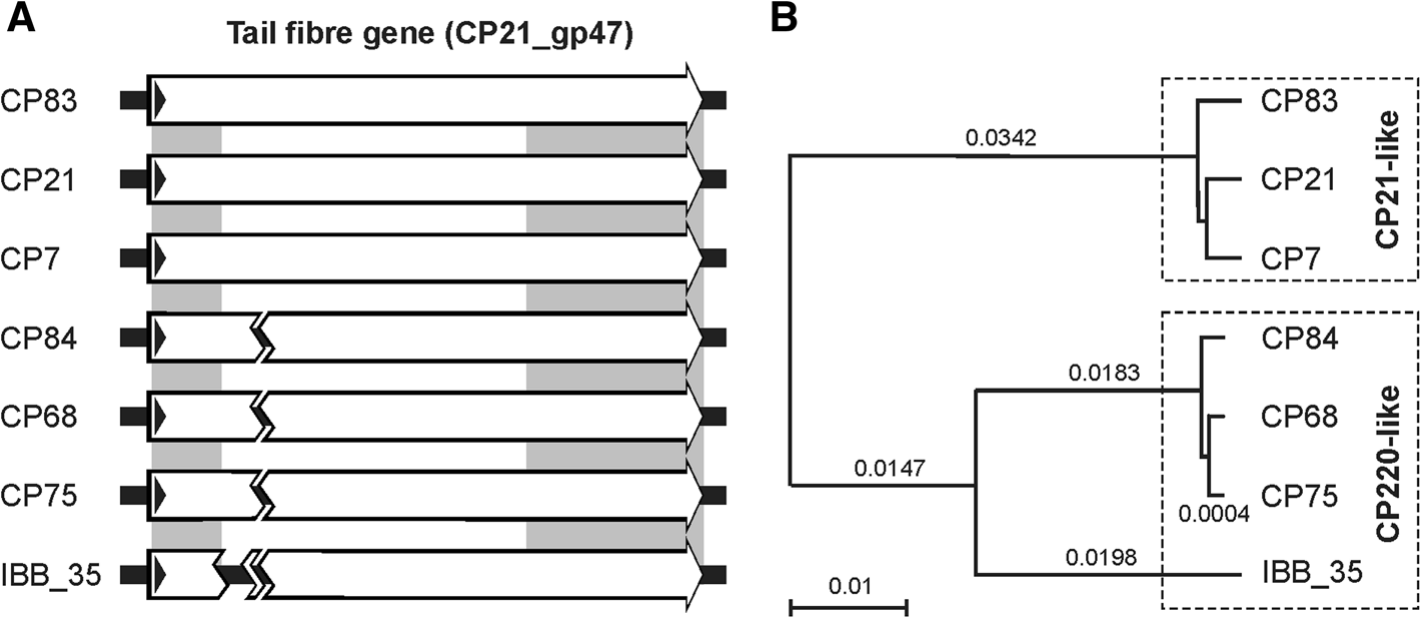
Similarities of the tail fiber genes. **a** Schematic illustration of the genes indicating the deletion that was found in the CP220 subgroup. **b** Dendrogram showing the similarities of the tail fiber genes

The previous studies demonstrated that many structural genes of the phages, some of which encoding tail proteins are differently arranged in the two subgroups. Even though the inversion of the modules B and C did not affect the integrity of the respective genes, it cannot be excluded that the genomic rearrangement resulted in a change of phenotypic properties. To address this issue, we determined the host specificity of members of each subgroup. For this study, 255 *Campylobacter* spp*.* (i.e., *C. coli*, *C. jejuni*, *C. lari*, *C. fetus*, *C. sputorum* and *C. hyointestinalis*) strains were investigated in terms of phage susceptibility (plaque formation). As shown in Table 1, phages belonging to the CP220/CPt10 subgroup lysed a significant higher number of strains than members of the CP21 subgroup. Moreover, phages of each subgroup exhibited similar host ranges. They mostly lysed the same strains (see in Additional file 1: Table S1). Thus, there was a striking coincidence between the modular organization and the host specificity of group II phages. The only exception was IBB_35 which lysed a high proportion of *C. coli* strains but, similar to phages of the CP21 subgroup, only few *C. jejuni* strains. Gene product 047 (gp047) of *Campylobacter* phages has already been identified as a receptor binding protein [24]. The host recognition domains are localized in the conserved C-terminus of gp047. To elucidate whether variations within gp047 may account for the observed differences in host specificity, we analysed gp047 nucleotide sequences of the group II phages. Compared to members of the CP21 subgroup, all phages belonging to the CP220 subgroup revealed a deletion (0.2 to 2.5 kb) within gp047, (Fig. 10a). In addition, the region encoding the C-terminus of Gp047 was more similar within each subgroup than to members of the other subgroup (Fig. 10b). Only gp047 of IBB_35 showed some sequence deviations, which might be responsible for the distinct host range of this phage. The data indicate that the subgroups do not only differ in their genome organization but also in particular genes. This finding was corroborated by a PCR analysis of tRNA genes. As with CP21 all members of its subgroup revealed tRNA genes for threonine and proline, while phages belonging to the CP220 subgroup possess tRNA genes for arginine and tyrosine.

## Discussion

Over the last decade an increasing number of reports have been published describing the reduction of *Campylobacter* in chicken and on food products by application of lytic phages [1, 4–16, 21]. One prerequisite for a commercial exploitation of phages is a profound knowledge on their biology and genetics. Thus far, a number of *Campylobacter* group II and group III phages has been genetically characterized [24]. All phages of the same group are closely related to each other while at the nucleotide level, there is only limited similarity between phages belonging to different groups [24, 25]. The genomic analysis of CP21 confirmed the strong and more distant relationship to other group II and group III phages, respectively. However, the study also disclosed that group II phages are not uniform in terms of their gene content and genome organization. While group III phages possess numerous homing endonucleases that might be important for reorganization of genes [25], homing endonucleases are less frequently found in group II phages. Though, group II phages encode transposases which similarly might be involved in genetic rearrangements. CP21 e.g., codes for five putative transposases, and in CP220 four transposase genes have been detected. In addition, the alignment of the CP21 and CP220 genomes revealed a number of sites where transposase activities may have caused deletions [19].

Besides these small-scale changes, group II phages have obviously also used shuffling of larger DNA fragments resulting in more pronounced genomic variations. The detailed analysis of the repeat regions of CP21 and CP220/CPt10 showed that all of them contain the same core consensus sequence that we also found in IBB_35. Moreover, adjacent sequences inside each unit are also related to each other. Therefore, modular shuffling might have occurred both within and between group II phage genomes. The striking diversity of the number and orientation of units within each repeat region points to previous recombination events. Notable is the fact that the analysed group II phages additionally contain single repeat units or the 13 bp core sequence on their genomes. A massive occurrence of repetitive sequences as is found in CP21, CP220 and CPt10 is rather uncommon for phages. In *Tsukamurella* phage TPA2 a large number of inverted repeats have been detected but the function of these repeat structures is unknown since neither replication origins nor transposable elements could be identified on the TPA2 genome [42].

In this study, a PCR was developed, by use of which the modular organization of the CP21 genome has been confirmed. Our PCR and restriction analyses of yet not sequenced group II phages suggest that these are similarly composed of modules. This also pertains to phage IBB_35 where previous attempts failed to join five single contigs that correspond well with the modules of CP220/CPt10 [22]. It should be emphasized that in contrast to the analysis of phage CP220 [11], we did not obtain amplicons of mixed lengths in our PCR experiments. One important difference of the studies is that some of our primers were approximately twice as long as those used with CP220. Due to the very high A + T content of the phage DNAs and secondary structures probably formed by the repeat regions, it is likely that the long primers were bound more specifically avoiding the synthesis of by products. As it can be seen in Fig. 7a, some PCR reactions showed a smear but we never observed a ladder of amplicons as it was shown for CP220 [11]. Hence, it is questionable whether group II phages actually possess genomes of variable length.

So far, only two subgroups of group II phages possessing distinct modular arrangements have been identified. Due to the wide distribution of the repeat core sequence on the phage genomes further subgroups might exist. Though, the currently available data suggest that the subgroups did not evolve recently because members of each subgroup do not only possess the same composition of modules but also the same tRNAs and similar tail fiber genes. Moreover, a sequence analysis of the tail tube gene of the phages revealed a number of positions with subgroup-specific nucleotides (see in Additional file 1: Table S5). This points to a more ancient split of group II phages. It is of course also possible that other modular compositions may have dramatically consequences since they might affect the transcription of genes which can disrupt the integrity of the phages yielding defective particles. The inversion of the modules B and C in CP21 resulted in a reorganization of structural genes but this variation had apparently no influence on the activity of the phage. Yet, the subgroups differed significantly in their host range. Even though there were some minor divergencies within each subgroup, their members showed very similar lysis patterns. Whether the host specificity of the subgroups is mainly determined by the modular genome organization, by sequence variations within single products like gp047, by both or by other factors has still to be unravelled. Nevertheless, we found a clear link between the host range of particular phages, their modular genome composition and sequence polymorphisms of gp047. The exception to this rule was phage IBB_35. On the one hand it possesses general properties similar to other group II phages. This also refers to the behaviour of the DNA during phenol extraction. Partitioning into the interphase, how we observed it with group III phages [25] did not occur with IBB_35. On the other hand, however, there was a discrepancy between the modular genome organisation of IBB_35, which is the same as in phages of the CP220 subgroup, and the host range of the phage. IBB_35 lysed significantly less *C. jejuni* strains than e.g., CP84. Moreover, IBB_35 revealed distinct VspI and SmiI restriction pattern and its gp047 and tail tube gene showed a number of nucleotide exchanges in relation to other phages of the CP220 subgroup. We therefore conclude that IBB_35 represents a more distantly related member of this subgroup.

## Conclusion

In summary the presented data demonstrate that *Campylobacter* group II phages are diverse regarding their genome organization. The currently identified subgroups are at least also different in terms of their tail fiber, tail tube and tRNA genes. It is conceivable that the subgroups evolved a long time ago and that they do not undergo further changes. Virulence-associated genes have yet not been detected in *Campylobacter* phages. Whether they are genetically stable, which would be another important prerequisite for their exploitation, has still to be elucidated by further experiments focussing on the question which role homing endonucleases and transposases may play for genetic variations, e.g., the acquisition of foreign genes. Sequencing of more group II phage genomes would additionally be conducive to answer the question, whether these phages are suitable and save.

### Availability of supporting data

Data sets supporting the results of this article are included within the article and its additional files. The genome sequence determined in this study has been deposited in the National Center for Biotechnology Information (NCBI) Genbank database under accession number HE815464. Access to the data is available upon publication at http://www.ncbi.nlm.nih.gov/nuccore/.

## Additional file

Additional file 1: Table S1. Strain specificity of *Campylobacter* phages. **Table S2.** CP21 DNA regions that are absent or relocated in the other group II phages. **Table S3.** CP21 ORF analysis. **Table S4.** Primers used in this study. **Table S5.** Nucleotide differences within the group II tail tube genes. **Figure S1.** Additional repeat region on the genomes of CP21, CP220 and CPt10. Dot plot analyses of the additional repeat region of CP21 (A), CP220 (B) and CPt10 (C). (DOCX 288 kb)

## Abbreviations

BfR: Bundesinstitut für Risikobewertung (Federal Institute for Risk Assessment)
CNET: Campynet
CsCl: Caesium chloride
DSMZ: German collection of microorganisms and cell cultures
gp: Gene product
IBB_35: vB_CcoM-IBB_35
NCTC: National collection of type cultures
OD: Optical density
ORF: Open Reading Frame
LB: Lysogeny broth
PTP: Picotiterplate
RR: Repeat regions
SM-buffer: Sodium chloride/magnesium sulfate-buffer
TEM: Transmission electron microscopy
wt/vol: Weight per volume

## References

[1] Carvalho CM, Gannon BW, Halfhide DE, Santos SB, Hayes CM, Roe JM (2010). The in vivo efficacy of two administration routes of a phage cocktail to reduce numbers of *Campylobacter coli* and *Campylobacter jejuni* in chickens. BMC Microbiol.

[2] Lee MD, Newell DG (2006). *Campylobacter* in poultry: filling an ecological niche. Avian Dis.

[3] Humphrey T, O’Brien S, Madsen M (2007). Campylobacters as zoonotic pathogens: a food production perspective. Int J Food Microbiol.

[4] Connerton PL, Timms AR, Connerton IF (2011). *Campylobacter* bacteriophages and bacteriophage therapy. J Appl Microbiol.

[5] Atterbury RJ, Connerton PL, Dodd CE, Rees CE, Connerton IF (2003). Application of host-specific bacteriophages to the surface of chicken skin leads to a reduction in recovery of *Campylobacter jejuni*. Appl Environ Microbiol.

[6] Connerton PL, Loc-Carrillo CM, Swift C, Dillon E, Scott A, Rees CE (2004). Longitudinal study of *campylobacter jejuni* bacteriophages and their hosts from broiler chickens. Appl Environ Microbiol.

[7] El-Shibiny A, Scott A, Timms A, Metawea Y, Connerton PL, Connerton IF (2009). Application of a group II *campylobacter* bacteriophage to reduce strains of *campylobacter jejuni* and *campylobacter coli* colonizing broiler chickens. J Food Prot.

[8] Gibbens JC, Pascoe SJ, Evans SJ, Davies RH, Sayers AR (2001). A trial of biosecurity as a means to control *campylobacter* infection of broiler chickens. Prev Vet Med.

[9] Hwang S, Yun J, Kim KP, Heu S, Lee S, Ryu S (2009). Isolation and characterization of bacteriophages specific for *campylobacter jejuni*. Microbiol Immunol.

[10] Loc-Carrillo CM, Atterbury RJ, El-Shibiny A, Connerton PL, Dillon E, Scott A (2005). Bacteriophage therapy to reduce *campylobacter jejuni* colonization of broiler chickens. Appl Environ Microbiol.

[11] Timms AR, Cambray-Young J, Scott AE, Petty NK, Connerton PL, Clarke L (2010). Evidence for a lineage of virulent bacteriophages that target *campylobacter*. BMC Genomics.

[12] Wagenaar J, Jacobs-Reitsma W, Hofshagen M, Newell D, Nachamkin I (2008). Poultry colonization with *campylobacter* and its control at the primary production level. Campylobacter.

[13] Wagenaar JA, van Bergen MA, Mueller MA, Wassenaar TM, Carlton RM (2005). Phage therapy reduces *campylobacter jejuni* colonization in broilers. Vet Microbiol.

[14] Fischer S, Kittler S, Klein G, Glunder G (2013). Impact of a single phage and a phage cocktail application in broilers on reduction of *campylobacter jejuni* and development of resistance. PLoS One.

[15] Kittler S, Fischer S, Abdulmawjood A, Glunder G, Klein G (2013). Effect of bacteriophage application on *Campylobacter jejuni* loads in commercial broiler flocks. Appl Environ Microbiol.

[16] Kittler S, Fischer S, Abdulmawjood A, Glunder G, Klein G (2014). Colonisation of a phage susceptible *Campylobacter jejuni* population in two phage positive broiler flocks. PLoS One.

[17] Endersen L, O’Mahony J, Hill C, Ross RP, McAuliffe O, Coffey A (2014). Phage therapy in the food industry. Annu Rev Food Sci Technol.

[18] Hagens S, Loessner MJ (2010). Bacteriophage for biocontrol of foodborne pathogens: calculations and considerations. Curr Pharm Biotechnol.

[19] Hammerl JA, Jaeckel C, Hertwig S (2014). Genetik von *Campylobacter* Phagen. Berliner Münchner Tierärztliche Wochenschrift.

[20] Sails AD, Wareing DR, Bolton FJ, Fox AJ, Curry A (1998). Characterisation of 16 *Campylobacter jejuni* and *C. coli* typing bacteriophages. J Med Microbiol.

[21] Hammerl JA, Jaeckel C, Alter T, Janzcyk P, Stingl K, Knüver M (2014). Reduction of *Campylobacter jejuni* in broiler chicken by successive application of groupII and groupIII phages. PLoS One.

[22] Carvalho CM, Kropinski AM, Lingohr EJ, Santos SB, King J, Azeredo J (2012). The genome and proteome of a *Campylobacter coli* bacteriophage vB_CcoM-IBB_35 reveal unusual features. Virol J.

[23] Hammerl JA, Jackel C, Reetz J, Hertwig S (2012). The complete genome sequence of bacteriophage CP21 reveals modular shuffling in *Campylobacter* group II phages. J Virol.

[24] Javed MA, Ackermann HW, Azeredo J, Carvalho CM, Connerton I, Evoy S (2014). A suggested classification for two groups of *Campylobacter* myoviruses. Arch Virol.

[25] Hammerl JA, Jaeckel C, Reetz J, Beck S, Alter T, Lurz R (2011). *Campylobacter jejuni* group III phage CP81 contains many T4-like genes without belonging to the T4-type phage group: implications for the evolution of T4 phages. J Virol.

[26] Lima-Mendez G, Toussaint A, Leplae R (2011). A modular view of the bacteriophage genomic space: identification of host and lifestyle marker modules. Res Microbiol.

[27] Botstein D (1980). A theory of modular evolution for bacteriophages. Ann N Y Acad Sci.

[28] Hendrix RW (1999). Evolution: the long evolutionary reach of viruses. Curr Biol.

[29] Hendrix RW, Lawrence JG, Hatfull GF, Casjens S (2000). The origins and ongoing evolution of viruses. Trends Microbiol.

[30] Juhala RJ, Ford ME, Duda RL, Youlton A, Hatfull GF, Hendrix RW (2000). Genomic sequences of bacteriophages HK97 and HK022: pervasive genetic mosaicism in the lambdoid bacteriophages. J Mol Biol.

[31] Hansen VM, Rosenquist H, Baggesen DL, Brown S, Christensen BB (2007). Characterization of *Campylobacter* phages including analysis of host range by selected *Campylobacter* Penner serotypes. BMC Microbiol.

[32] Sambrook J, Russel D (2001). Molecular cloning: a laboratory manual.

[33] Hertwig S, Klein I, Schmidt V, Beck S, Hammerl JA, Appel B (2003). Sequence analysis of the genome of the temperate *Yersinia enterocolitica* phage PY54. J Mol Biol.

[34] Luo C, Tsementzi D, Kyrpides N, Read T, Konstantinidis KT (2012). Direct comparisons of Illumina vs. Roche 454 sequencing technologies on the same microbial community DNA sample. PLoS One.

[35] Aziz RK, Bartels D, Best AA, DeJongh M, Disz T (2008). The RAST Server: rapid annotations using subsystems technology. BMC Genomics.

[36] Glass EM, Wilkening J, Wilke A, Antonopoulos D, Meyer F (2010). Using the metagenomics RAST server (MG-RAST) for analyzing shotgun metagenomes. Cold Spring Harb Protoc.

[37] Meyer F, Paarmann D, D’Souza M, Olson R, Glass EM, Kubal M (2008). The metagenomics RAST server - a public resource for the automatic phylogenetic and functional analysis of metagenomes. BMC Bioinformatics.

[38] Altschul SF, Madden TL, Schaffer AA, Zhang J, Zhang Z, Miller W (1997). Gapped BLAST and PSI-BLAST: a new generation of protein database search programs. Nucleic Acids Res.

[39] Ermolaeva MD, Khalak HG, White O, Smith HO, Salzberg SL (2000). Prediction of transcription terminators in bacterial genomes. J Mol Biol.

[40] Bichara M, Wagner J, Lambert IB (2006). Mechanisms of tandem repeat instability in bacteria. Mutat Res.

[41] Chen F, Liu WQ, Eisenstark A, Johnston RN, Liu GR, Liu SL (2010). Multiple genetic switches spontaneously modulating bacterial mutability. BMC Evol Biol.

[42] Petrovski S, Seviour RJ, Tillett D (2011). Genome sequence and characterization of the *Tsukamurella* bacteriophage TPA2. Appl Environ Microbiol.

