# Supplementary material for: Campylobacter group II phage CP21 is the prototype of a new subgroup revealing a distinct modular genome organization and host specificity
Source: BMC Genomics. 2015 Aug 22;16(1):629. doi: 10.1186/s12864-015-1837-1 (PMC4546147; doi:10.1186/s12864-015-1837-1)

**Additional file 1**

**Table S1. Strain specificity of *Campylobacter* phages**

| **Strain** | **Origin**  **(country)** | **Source** | **LEP type** | **Penner type** | **Phage type** | **Fla type** | **CP68** | **CP75** | **CP84** | **CP7** | **CP83** | **CP21** | **IBB35** |
| --- | --- | --- | --- | --- | --- | --- | --- | --- | --- | --- | --- | --- | --- |
| ***Campylobacter coli*** | | | | | | | | | | | | | |
| 12668 | UK | Unkown | n.d. | n.d. | n.d. | n.d. | + | + | + | + | + | + | + |
| CNET064 | Denmark | Chicken | 51 | 54 | 44 | 7 | + | + | + | + | - | + | + |
| CNET068 | Northern Ireland | Pig | 26 | ^(A)^ | 2 | 4 | + | + | + | + | - | - | - |
| CNET051 | France | Human | 59 | 19, 24 | 44 | 21 | + | + | + | - | - | - | - |
| CNET021 | The Netherlands | Chicken | 11 | 58 | 44 | 21 | + | + | + | - | - | - | - |
| CNET020 | The Netherlands | Chicken | 11 | 58 | 44 | 21 | + | + | + | - | - | - | - |
| BfR9026 | Germany | Chicken | n.d. | n.d. | n.d. | n.d. | + | + | + | - | - | - | + |
| BfR9031 | Germany | Turkey | n.d. | n.d. | n.d. | n.d. | + | + | + | - | - | - | + |
| BfR9036 | Germany | Turkey | n.d. | n.d. | n.d. | n.d. | + | + | + | + | + | + | + |
| BfR9040 | Germany | Turkey | n.d. | n.d. | n.d. | n.d. | + | + | + | + | - | + | + |
| ***Campylobacter jejuni*** | | | | | | | | | | | | | |
| 12662 | UK | Human | n.d. | n.d. | n.d. | n.d. | + | + | + | + | + | + | + |
| CNET060 | United Kingdom | Sheep | 11 | 5, 6 | 44 | 39 | + | + | + | - | - | - | - |
| CNET012 | Northern Ireland | Human | 8 | 2 | 35 | 28 | + | + | + | - | - | + | - |
| CNET038 | Belgium | Human | 19 | 19 | 2 | 2 | + | + | + | - | - | - | - |
| CNET049 | France | Human | ^(A)^ | 58 | 1 | 5 | + | + | + | - | - | - | - |
| CNET024 | Finland | Wildbird | 9 | 7, 15 | 1 | 42 | + | - | - | + | - | - | - |
| CNET056 | Denmark | Cattle | 45 | ^(A)^ | 2 | 24 | + | + | + | - | - | - | - |
| CNET099 | United Kingdom | Human | 6 | 58 | 1 | 40 | - | - | + | + | + | + | - |
| CNET112 | Sweden | Canin | 50 | 42 | 5 | 6 | + | + | + | + | + | + | - |
| CNET100 | United Kingdom | Human | 6 | ^(A)^ | 39 | 40 | - | - | + | + | + | + | - |
| CNET095 | Denmark | Chicken | 50 | 58 | 2 | 17 | + | - | + | - | + | - | - |
| CNET028 | Finland | Wildbird | 18 | 58 | 66 | 16 | + | + | + | + | + | + | - |
| BfR7832 | Germany | Chicken | n.d. | n.d. | n.d. | n.d. | + | + | + | - | - | - | - |
| BfR7911 | Germany | Chicken | n.d. | n.d. | n.d. | n.d. | + | + | + | + | + | + | + |
| BfR7939 | Germany | Milk | n.d. | n.d. | n.d. | n.d. | - | - | + | - | - | - | - |
| BfR8076 | Germany | Chicken | n.d. | n.d. | n.d. | n.d. | + | + | + | + | + | + | - |
| BfR8457 | Germany | Pigeon | n.d. | n.d. | n.d. | n.d. | + | + | + | - | - | - | + |
| BfR8540 | Germany | Turkey | n.d. | n.d. | n.d. | n.d. | + | + | + | - | - | - | + |
| BfR8592 | Germany | Goose | n.d. | n.d. | n.d. | n.d. | + | + | + | - | - | - | - |
| BfR8656 | Germany | Chicken | n.d. | n.d. | n.d. | n.d. | - | - | - | + | - | - | - |
| BfR8697 | Germany | Turkey | n.d. | n.d. | n.d. | n.d. | + | + | - | + | + | + | + |
| BfR8887 | Germany | Duck | n.d. | n.d. | n.d. | n.d. | - | - | - | + | - | - | - |
| BfR8890 | Germany | Chicken | n.d. | n.d. | n.d. | n.d. | - | - | + | - | + | + | - |
| BfR8919 | Germany | Turkey | n.d. | n.d. | n.d. | n.d. | + | + | + | - | - | - | - |
| BfR8930 | Germany | Duck | n.d. | n.d. | n.d. | n.d. | + | + | + | + | + | + | - |
| BfR8933 | Germany | Turkey | n.d. | n.d. | n.d. | n.d. | - | + | + | - | - | - | - |
| RM1221 | UK | Chicken | n.d. | n.d. | n.d. | n.d. | + | + | + | + | + | + | + |

(A), untypable; n.d., not determined

**Table S2. CP21 DNA regions that are absent or relocated in the other group II phages**

| **Locus No.** | **CP21 position** | **Size (kb)** | **Predicted function (CP21)** | **Presence in *Campylobacter* phage: ^(1)^** | | |
| --- | --- | --- | --- | --- | --- | --- |
|  |  |  |  | **CP220** | **CPt10** | **IBB_35** |
| 1 | 9.8-11.6 kb | ~1.8 | adenine-specific methyltransferase | - | + (98%) | - |
| 2 | 22.3-22.7 kb | ~0.4 | hypothetical protein  transposase | + (94%) | - | - |
| 3 | 30.7-32.5 kb | ~1.8 | tryptophan halogenase,  hypothetical protein | - | - | + (91%) |
| 4 | 52.5-52.7 kb | ~0.2 | hypothetical protein | + (90%) | + (91%) | + (91%) |
| 5 | 60.3-62.0 kb | ~1.7 | transposase | + (82%) | + (83%) | + (82%) |
| 6 | 68.6-69.9 kb | ~1.3 | hypothetical proteins | - | - | - |
| 7 | 72.0-72.4 kb | ~2.4 | hypothetical protein | - | + (95%) | + (95%) |
| 8 | 74.6-75.7 kb | ~1.1 | hypothetical proteins | - | - | - |
| 9 | 85.5-87.2 kb | ~1.7 | hypothetical protein,  DNA polymerase | - | - | - |
| 10 | 118.5-119.5 kb | ~1.0 | hypothetical protein | (+) (99%) | + (97%) | + (95%) |
| 11 | 122.0-125.0 kb | ~3.0 | N6-adenosine-methyltransferase | - | (+) (91%) | (+) (93%) |
| 12 | 126.5-128.5 kb | ~2.0 | Transposases | - | + (98%) | - |
| 13 | 129.0-131.5 kb | ~2.5 | Transposases | (+) (95%) | (+) (95%) | (+) (95%) |
| 14 | 135.5-138.5 kb | ~3.0 | homing endonucleases |  |  | (+) (96%) |
| 15 | 138.5-140.5 kb | ~2.0 | hypothetical protein | - | - | - |
| 16 | 141.7-142.8 kb | ~1.1 | Gp18-like tail sheath protein | - | - | - |
| 17 | 158.2-160.7 kb | ~2.5 | tail fibre proteins | - | - | + (96%) |
| 18 | 162.2-162.6 kb | ~0.4 | hypothetical protein | - | - | - |

^(1^) Percentages of BlastN nucleotide sequence identity values are given in brackets.

**Table S3. CP21 ORF analysis**

| **ORF No.** | **Strand** | **Start** | **Stop** | **Length (bp)** | **Length (aa)** | **CP220** | **CPt10** | **IBB35** | **12673** | **CPX** | **CP81** | **Predicted function** | **Best match (NCBI - Blastp search)** | **E-value** | **Identities** | **Accession number** |
| --- | --- | --- | --- | --- | --- | --- | --- | --- | --- | --- | --- | --- | --- | --- | --- | --- |
| ORF001 | + | 93 | 1304 | 1212 | 403 | 97% | 97% | - | - | 30% | 30% | Phage DNA packaging protein terminase (T4 gp17-like) | Phage DNA packaging protein (terminase) [*Campylobacter* phage CP220] | 0.0 | 355/367 (97%) | CBJ93810.1 |
| ORF002 | + | 1273 | 2538 | 1266 | 421 | - | - | 89% | - | - | - | Unknown | Hypothetical protein [*Campylobacter* phage vB_CcoM-IBB_35] | 0.0 | 375/421 (89%) | AEI88228.1 |
| ORF003 | + | 2535 | 4379 | 1845 | 614 | 90% | 90% | 91% | - | 33% | 33% | DNA packaging protein (terminase) (T4 gp17-like) | Phage DNA packaging protein (terminase) [*Campylobacter* phage CP220] | 0.0 | 368/403 (91%) | CBJ93810.1 |
| ORF004 | + | 4395 | 5123 | 729 | 242 | 97% | 96% | 97% | - | - | - | Base plate protein (T4 gp51-like) | gp51 base plate protein [*Campylobacter* phage vB_CcoM-IBB_35] | 8,00E-162 | 232/240 (97%) | AEI88226.1 |
| ORF005 | - | 5577 | 5158 | 420 | 139 | 91% | 94% | 92% | - | - | - | Unknown | Hypothetical phage protein [*Campylobacter* phage CPt10] | 9,00E-86 | 131/139 (94%) | CBJ94205.1 |
| ORF006 | + | 5959 | 7473 | 1515 | 504 | 99% | 99% | 98% | 33% | - | - | DNA topoisomerase II | Phage DNA topoisomerase (large subunit) [*Campylobacter* phage CPt10] | 0.0 | 498/503 (99%) | CBJ94207.1 |
| ORF007 | + | 7470 | 8003 | 534 | 177 | 94% | 94% | 95% | - | - | - | Unknown | Hypothetical phage protein [*Campylobacter* phage CP220] | 1,00E-117 | 167/177 (94%) | CBJ93815.1 |
| ORF008 | + | 8015 | 8473 | 459 | 152 | 97% | 97% | 95% | 43% | 44% | 42% | dUTP pyrophosphatase | Phage protein (possible dUTP pyrophosphatase) [*Campylobacter* phage CP220] | 2,00E-103 | 148/152 (97%) | CBJ93816.1 |
| ORF009 | + | 8515 | 8757 | 243 | 80 | 98% | 98% | 99% | - | - | - | Unknown | Hypothetical protein [*Campylobacter* phage vB_CcoM-IBB_35] | 2,00E-50 | 79/80 (99%) | AEI88220.1 |
| ORF010 | + | 8770 | 9846 | 1077 | 358 | 93% | 98% | 91% | 28% | 28% | 28% | Primase (T4 gp61-like) | DNA primase subunit [*Campylobacter* phage CPt10] | 0.0 | 350/358 (98%) | CBJ94211.1 |
| ORF011 | + | 9843 | 11453 | 1611 | 536 | - | - | - | 80% | 80% | 89% | Adenine specific DNA methyltransferase | Putative DNA methylase [*Campylobacter* phage CPX] | 0.0 | 301/377 (80%) | YP_004956907.1 |
| ORF012 | + | 11631 | 12545 | 915 | 304 | 98% | 98% | 96% | 38% | 37% | 38% | Sliding clamp loader - DNA polymerase accessory factor (T4 gp44-like) | DNA polymerase accessory factor (sliding clamp loader) [*Campylobacter* phage CPt10] | 4,00E-174 | 298/304 (98%) | CBJ94213.1 |
| ORF013 | + | 12555 | 13613 | 1059 | 352 | 99% | 99% | 99% | 28% | 26% | 26% | RNaseH | Phage RNase H [*Campylobacter* phage CP220] | 0.0 | 349/352 (99%) | CBJ93820.1 |
| ORF014 | + | 13676 | 14224 | 549 | 182 | 86% | 85% | 85% | - | - | - | Unknown | Hypothetical phage protein [*Campylobacter* phage CP220] | 2,00E-108 | 156/182 (86%) | CBJ93821.1 |
| ORF015 | + | 14269 | 14901 | 633 | 210 | 96% | 95% | 94% | - | - | - | Minor tail protein | Hypothetical phage protein [*Campylobacter* phage CP220] | 6,00E-143 | 201/210 (96%) | CBJ93822.1 |
| ORF016 | + | 14961 | 15638 | 678 | 225 | 93% | 95% | 95% | - | - | - | Base plate hub subunit protein | Baseplate hub subunit protein [*Campylobacter* phage CPt10] | 3,00E-153 | 213/225 (95%) | CBJ94217.1 |
| ORF017 | + | 15653 | 16345 | 693 | 230 | 97% | 94% | 93% | - | - | - | Unknown | Hypothetical phage protein [*Campylobacter* phage CP220] | 5,00E-160 | 221/227 (97%) | CBJ93824.1 |
| ORF018 | + | 16448 | 16666 | 219 | 72 | 81% | - | 82% | - | - | - | Unknown | Hypothetical protein [*Campylobacter* phage vB_CcoM-IBB_35] | 9,00E-34 | 59/72 (82%) | AEI88212.1 |
| ORF019 | + | 16769 | 17563 | 795 | 264 | 98% | 98% | 98% | - | - | - | Unknown | Hypothetical phage protein [*Campylobacter* phage CP220] | 0.0 | 260/264 (98%) | CBJ93826.1 |
| ORF020 | + | 17560 | 17823 | 264 | 87 | 91% | 98% | 92% | - | - | - | Unknown | Hypothetical phage membrane protein [*Campylobacter* phage CPt10] | 1,00E-46 | 84/86 (98%) | CBJ94220.1 |
| ORF021 | + | 17833 | 18603 | 771 | 256 | 96% | 97% | 92% | - | - | - | UDP-glucose dehydrogenase | Hypothetical phage protein [*Campylobacter* phage CPt10] | 2,00E-142 | 249/256 (97%) | CBJ94221.1 |
| ORF022 | + | 18628 | 19005 | 378 | 125 | 94% | - | 85% | - | - | - | Unknown | Hypothetical phage protein [*Campylobacter* phage CP220] | 8,00E-79 | 118/125 (95%) | CBJ93829.1 |
| ORF023 | + | 19005 | 19943 | 939 | 312 | 98% | 97% | 97% | - | - | - | Unknown | Hypothetical phage protein [*Campylobacter* phage CP220] | 0.0 | 306/312 (98%) | CBJ93830.1 |
| ORF024 | + | 19921 | 20145 | 225 | 74 | 97% | 60% | 82% | - | - | - | Unknown | Hypothetical phage protein [*Campylobacter* phage CP220] | 2,00E-42 | 72/74 (97%) | CBJ93831.1 |
| ORF025 | + | 20147 | 21250 | 1104 | 367 | 99% | 99% | 99% | - | - | - | Radical SAM domain-containing protein | Hypothetical phage protein [*Campylobacter* phage CP220] | 0.0 | 366/367 (99%) | CBJ93832.1 |
| ORF026 | + | 21333 | 21665 | 333 | 110 | 95% | 92% | 98% | - | - | - | Clamp-loader subunit (T4 gp62-like) | gp62 clamp-loader subunit [*Campylobacter* phage vB_CcoM-IBB_35] | 5,00E-72 | 108/110 (98%) | AEI88204.1 |
| ORF027 | - | 22298 | 21693 | 606 | 201 | 99% | 99% | 99% | 38% | 38% | 38% | Unknown | Hypothetical protein [*Campylobacter* phage vB_CcoM-IBB_35] | 1,00E-143 | 199/201 (99%) | AEI88203.1 |
| ORF028 | - | 22532 | 22365 | 168 | 55 | 93% | - | - | - | - | - | ISCaje4 transposase TnpB | ISCaje4 transposase TnpB [Campylobacter phage CP220] | 2,00E-25 | 51/55 (93%) | CBJ93955.1 |
| ORF029 | + | 22702 | 24213 | 1512 | 503 | 99% | 99% | 99% | - | - | - | Unknown | Hypothetical phage protein [*Campylobacter* phage CP220] | 0.0 | 500/503 (99%) | CBJ93835.1 |
| ORF030 | + | 24239 | 25426 | 1188 | 395 | 95% | 94% | 92% | - | - | - | Virion structural protein | Possible virion structural protein [*Campylobacter* phage CP220] | 0.0 | 377/395 (95%) | CBJ93836.1 |
| ORF031 | + | 25470 | 25640 | 171 | 56 | 87% | 98% | 89% | - | - | - | hypothetical phage membrane protein | Hypothetical phage membrane protein (*Campylobacter* phage CPt10) | 2,00E-29 | 54/55(98%) | CBJ94230.1 |
| ORF032 | + | 25752 | 26192 | 441 | 146 | 95% | 99% | 97% | - | - | - | EndoVII packaging and recombination endonuclease | EndoVII packaging and recombination endonuclease [*Campylobacter* phage CPt10] | 4,00E-89 | 143/144 (99%) | CBJ94231.1 |
| ORF033 | + | 26219 | 27898 | 1680 | 559 | 99% | 99% | 99% | 32% | 32% | 32% | gp20 portal vertex protein of head | Possible phage prohead assembly initiator protein/portal protein [*Campylobacter* phage CPt10] | 0.0 | 514/516(99%) | CBJ94232.1 |
| ORF034 | + | 27992 | 28510 | 519 | 172 | 56% | 45% | 47% | - | - | - | Unknown | Hypothetical protein [*Campylobacter fetus* subsp. *fetus*] | 3,00E-29 | 76/170 (45%) | CBH51834.1 |
| ORF035 | + | 28575 | 29429 | 855 | 284 | - | - | - | - | - | - | Unknown | Hypothetical protein [*Campylobacter* phage vB_CcoM-IBB_35] | 3,00E-50 | 112/175 (64%) | AEI88196.1 |
| ORF036 | + | 29848 | 30738 | 891 | 296 | 99% | 99% | 97% | 28% | 29% | 29% | DNA ligase (T4 gp30-like) | Phage DNA ligase [*Campylobacter* phage CP220] | 0.0 | 295/296 (99%) | CBJ93842.1 |
| ORF037 | + | 30748 | 31947 | 1200 | 399 | - | - | 92% | - | - | - | Tryptophan halogenase | Putative tryptophan halogenase [*Campylobacter* phage vB_CcoM-IBB_35] | 0.0 | 366/399 (92%) | AEI88194.1 |
| ORF038 | + | 31944 | 32069 | 126 | 41 | - | - | 76% | - | - | - | Unknown | Hypothetical protein [*Campylobacter* phage vB_CcoM-IBB_35] | 1,00E-11 | 29/38 (76%) | AEI88193.1 |
| ORF039 | - | 33988 | 32435 | 1554 | 517 | 97% | 99% | 97% | 25% | 25% | 25% | Tail sheath protein (T4 gp18-like) | Phage tail sheath protein [*Campylobacter* phage CPt10] | 0.0 | 507/514 (99%) | CBJ94236.1 |
| ORF040 | - | 35299 | 34097 | 1203 | 400 | 97% | 97% | 93% | - | - | - | Unknown | Hypothetical phage protein [*Campylobacter* phage CPt10] | 0.0 | 389/400 (97%) | CBJ94237.1 |
| ORF041 | - | 35725 | 35363 | 363 | 120 | 98% | 98% | 98% | - | - | - | Baseplate wedge subunit (T4 gp25-like) | gp25 baseplate wedge subunit [*Campylobacter* phage vB_CcoM-IBB_35] | 2,00E-81 | 118/120 (98%) | AEI88187.1 |
| ORF042 | - | 36217 | 35801 | 417 | 138 | 92% | 91% | 90% | 45% | 44% | 44% | Unknown | Hypothetical phage protein [Campylobacter phage CP220] | 2,00E-77 | 125/136(92%) | CBJ93847.1 |
| ORF043 | - | 36648 | 36319 | 330 | 109 | 90% | 87% | 88% | - | - | - | Unknown | Hypothetical phage exported protein [*Campylobacter* phage CP220] | 7,00E-62 | 97/108 (90%) | CBJ93848.1 |
| ORF044 | - | 36918 | 36706 | 213 | 70 | 84% | 91% | 93% | 42% | - | 42% | Membrane protein | Hypothetical phage membrane protein [*Campylobacter* phage CPt10] | 2,00E-40 | 64/70 (91%) | CBJ94241.1 |
| ORF045 | - | 37079 | 36927 | 153 | 50 | - | - | 96% | - | - | - | Unknown | Hypothetical protein [*Campylobacter* phage vB_CcoM-IBB_35] | 2,00E-25 | 48/50 (96%) | AEI88183.1 |
| ORF046 | + | 37169 | 40813 | 3645 | 1214 | 98% | 97% | 97% | 29% | 28% | 28% | Baseplate wedge protein (T4 gp6-like) | Phage baseplate wedge protein [*Campylobacter* phage CP220] | 0.0 | 1187/1214 (98%) | CBJ93850.1 |
| ORF047 | + | 40879 | 41550 | 672 | 223 | 86% | 99% | 98% | - | - | - | Unknown | Hypothetical protein [*Campylobacter* phage vB_CcoM-IBB_35] | 3,00E-157 | 217/222 (98%) | AEI88181.1 |
| ORF048 | + | 41561 | 41719 | 159 | 52 | 90% | 68% | 90% | - | - | - | Unknown | Hypothetical protein [*Campylobacter* phage vB_CcoM-IBB_35] | 3,00E-23 | 45/50 (90%) | AEI88180.1 |
| ORF049 | + | 41691 | 42209 | 519 | 172 | 96% | 92% | 96% | - | - | - | Unknown | Hypothetical phage protein [*Campylobacter* phage CP220] | 2,00E-91 | 163/170 (96%) | CBJ93852.1 |
| ORF050 | + | 42327 | 46427 | 4101 | 1366 | 97% | 97% | 95% | - | 27% | 27% | Unknown | Hypothetical phage protein [*Campylobacter* phage CPt10] | 0.0 | 1325/1366(97%) | CBJ94245.1 |
| ORF051 | + | 46443 | 47201 | 759 | 252 | 98% | 98% | 98% | 31% | 31% | 31% | Tail tube protein (T4 gp19-like) | Phage tail tube protein [*Campylobacter* phage CP220] | 0.0 | 246/252 (98%) | CBJ93854.1 |
| ORF052 | + | 47707 | 48132 | 426 | 141 | 98% | 99% | - | 37% | 36% | 36% | Head completion protein | Phage head completion protein [*Campylobacter* phage CPt10] | 6,00E-96 | 139/141 (99%) | CBJ94247.1 |
| ORF053 | + | 48333 | 48467 | 135 | 44 | - | - | - | - | - | - | Unknown | - | - | - | - |
| ORF054 | - | 49118 | 48570 | 549 | 182 | 93% | 94% | 91% | 51% | 51% | 53% | Thymidine kinase | Hypothetical phage protein [*Campylobacter* phage CP220] | 3,00E-121 | 169/182 (93%) | CBJ93967.1 |
| ORF055 | - | 50209 | 49193 | 1017 | 338 | 98% | 98% | 98% | - | - | - | Radical SAM domain-containing protein | Hypothetical phage protein (Radical SAM family) [*Campylobacter* phage CP220] | 0.0 | 332/338(98%) | CBJ93966.1 |
| ORF056 | - | 50419 | 50234 | 186 | 61 | 98% | 100% | 100% | - | - | - | Unknown | Hypothetical phage protein [*Campylobacter* phage CPt10] | 4,00E-37 | 61/61 (100%) | CBJ94365.1 |
| ORF057 | - | 50989 | 50444 | 546 | 181 | 99% | 94% | 94% | - | - | - | Phosphoesterase | Hypothetical phage protein [*Campylobacter* phage CP220] | 2,00E-125 | 176/178 (99%) | CBJ93964.1 |
| ORF058 | - | 51449 | 51024 | 426 | 141 | 94% | 84% | 89% | - | - | - | Unknown | Hypothetical phage protein [*Campylobacter* phage CPt10] | 7,00E-87 | 133/141 (94%) | CBJ94363.1 |
| ORF059 | - | 53786 | 51513 | 2274 | 757 | 91% | 92% | 97% | 27% | 26% | 26% | Unknown | Hypothetical phage protein [*Campylobacter* phage CPt10] | 0.0 | 696/755 (92%) | CBJ94362.1 |
| ORF060 | - | 54723 | 53923 | 801 | 266 | 96% | 98% | 98% | - | - | - | ClpP protease | Phage ClpP protease [*Campylobacter* phage CPt10] | 3,00E-151 | 260/264 (98%) | CBJ94361.1 |
| ORF061 | - | 56063 | 54735 | 1329 | 442 | 98% | 98% | 97% | - | - | - | DNA topoisomerase | Possible DNA topoisomerase (medium subunit) [*Campylobacter* phage CPt10] | 0.0 | 433/442 (98%) | CBJ94360.1 |
| ORF062 | - | 56856 | 56125 | 732 | 243 | 89% | 94% | 86% | - | - | - | hypothetical phage protein | Hypothetical phage protein [*Campylobacter* phage CPt10] | 3,00E-144 | 228/243 (94%) | CBJ94359.1 |
| ORF063 | - | 57295 | 56867 | 429 | 142 | 98% | 98% | 98% | - | - | - | Unknown | Hypothetical protein [*Campylobacter* phage vB_CcoM-IBB_35] | 2,00E-96 | 139/142 (98%) | AEF56842.1 |
| ORF064 | - | 59022 | 57604 | 1419 | 472 | 98% | 98% | 97% | 36% | 37% | 37% | ATP-dependent DNA/RNA helicase, uvsW | Phage ATP-dependent DNA/RNA helicase, uvsW [*Campylobacter* phage CP220] | 0.0 | 462/472 (98%) | CBJ93957.1 |
| ORF065 | - | 59708 | 59016 | 693 | 230 | 97% | 94% | 95% | 30% | - | - | RecB family exonuclease | Hypothetical phage protein [*Campylobacter* phage CP220] | 5,00E-161 | 222/230 (97%) | CBJ93956.1 |
| ORF066 | - | 59889 | 59722 | 168 | 55 | 92% | 98% | 96% | - | - | - | Unknown | Hypothetical phage protein [*Campylobacter* phage CPt10] | 5,00E-31 | 54/55 (98%) | CBJ94355.1 |
| ORF067 | - | 60391 | 59990 | 402 | 133 | 91% | 93% | 91% | - | - | - | Unknown | Hypothetical phage protein [*Campylobacter* phage CPt10] | 8,00E-85 | 124/133 (93%) | CBJ94354.1 |
| ORF068 | - | 61725 | 60409 | 1317 | 438 | 83% | 82% | 83% | - | - | - | ISCaje3 transposase | ISCaje3 transposase [*Campylobacter* phage CPt10] | 0.0 | 333/402 (83%) | CBJ93947.1 |
| ORF069 | - | 62052 | 61939 | 114 | 37 | - | - | 92% | - | - | - | Unknown | Hypothetical protein [*Campylobacter* phage vB_CcoM-IBB_35] | 5,00E-16 | 34/37 (92%) | AEF56847.1 |
| ORF070 | + | 62182 | 63096 | 915 | 304 | 95% | 97% | 96% | - | - | - | Unknown | Hypothetical phage protein [*Campylobacter* phage CPt10] | 9,00E-174 | 296/304 (97%) | CBJ94352.1 |
| ORF071 | - | 63446 | 63147 | 300 | 99 | 76% | 77% | 75% | - | - | - | Unknown | Hypothetical protein [*Campylobacter* phage vB_CcoM-IBB_35] | 1,00E-35 | 76/101 (75%) | AEF56849.1 |
| ORF072 | - | 63745 | 63494 | 252 | 83 | 90% | 89% | 92% | - | - | - | Unknown | Hypothetical phage membrane protein [*Campylobacter* phage CP220] | 4,00E-48 | 75/83 (90%) | CBJ93949.1 |
| ORF073 | - | 64310 | 63771 | 540 | 179 | 80% | 86% | 84% | - | - | - | Unknown | Hypothetical phage protein [*Campylobacter* phage CPt10] | 3,00E-100 | 154/179 (86%) | CBJ94349.1 |
| ORF074 | - | 64633 | 64448 | 186 | 61 | - | - | - | 80% | - | 83% | Unknown | Hypothetical protein [*Campylobacter* phage CP81] | 5,00E-10 | 38/46 (83%) | CBZ42225.1 |
| ORF075 | - | 65280 | 65038 | 243 | 80 | - | - | - | - | - | - | Unknown | - | - | - | - |
| ORF076 | - | 66506 | 65496 | 1011 | 336 | 98% | 99% | 99% | - | - | - | Radical SAM domain-containing protein | Hypothetical phage protein (Radical SAM family) [*Campylobacter* phage CPt10] | 0.0 | 300/303 (99%) | CBJ94347.1 |
| ORF077 | - | 67363 | 66533 | 831 | 276 | 99% | 97% | 99% | - | - | - | Unknown | Hypothetical protein [*Campylobacter* phage vB_CcoM-IBB_35] | 0.0 | 271/276 (98%) | AEI88242.1 |
| ORF078 | - | 68078 | 67401 | 678 | 225 | 96% | 92% | 93% | - | - | - | Unknown | Hypothetical protein [CP220] | 3,00E-138 | 216/225 (96%) | CBJ93944.1 |
| ORF079 | - | 68701 | 68201 | 501 | 166 | 100% | - | - | - | - | - | Ribonucleotide-diphosphate reductase | Probable phage ribonucleotide-diphosphate reductase alpha subunit [*Campylobacter* phage CP220] | 4,00E-100 | 153/153 (100%) | CBJ93943.1 |
| ORF080 | - | 68828 | 68691 | 138 | 45 | - | - | - | - | - | - | Unknown | - | - | - | - |
| ORF081 | - | 69015 | 68836 | 180 | 59 | - | - | - | - | - | - | Unknown | - | - | - | - |
| ORF082 | - | 69853 | 69035 | 819 | 272 | - | - | - | - | - | - | Unknown | - | - | - | - |
| ORF083 | - | 71988 | 69799 | 2190 | 729 | 98% | - | - | - | - | - | Ribonucleotide-diphosphate reductase | Phage ribonucleotide-diphosphate reductase alpha subunit [*Campylobacter* phage CP220] | 0.0 | 716/728 (98%) | CBJ93943.1\| |
| ORF084 | - | 72547 | 72089 | 459 | 152 | 93% | 91% | 93% | - | - | - | Unknown | Hypothetical protein [*Campylobacter* phage vB_CcoM-IBB_35] | 4,00E-92 | 137/147 (93%) | AEI88245.1 |
| ORF085 | + | 72679 | 73368 | 690 | 229 | 99% | 97% | 98% | - | - | - | Unknown | Hypothetical phage protein [*Campylobacter* phage CP220] | 1,00E-128 | 226/229 (99%) | CBJ93939.1 |
| ORF086 | + | 73389 | 73652 | 264 | 87 | 94% | 94% | 94% | - | - | - | Unknown | Hypothetical phage protein [*Campylobacter* phage CP220] | 3,00E-49 | 81/86 (94%) | CBJ93938.1 |
| ORF087 | + | 73662 | 75008 | 1347 | 448 | 76% | 79% | 94% | 60% | 60% | 60% | Unknown | Hypothetical phage protein [*Campylobacter* phage CP220] | 0.0 | 348/456 (76%) | CBJ93937.1 |
| ORF088 | + | 75139 | 75465 | 327 | 108 | - | - | - | - | - | - | Unknown | - | - | - | - |
| ORF089 | + | 75462 | 76088 | 627 | 208 | 91% | 85% | 76% | 58% | 57% | 57% | Unknown | Hypothetical phage protein [*Campylobacter* phage CPt10] | 3,00E-111 | 166/196 (85%) | CBJ94334.1 |
| tRNA-01 | - |  |  |  |  |  |  |  |  |  |  |  |  |  |  |  |
| ORF090 | - | 76824 | 76402 | 423 | 140 | 96% | 96% | 97% | - | - | - | Unknown | Hypothetical phage protein [*Campylobacter* phage CP220] | 7,00E-89 | 134/140 (96%) | CBJ93935.1 |
| tRNA-02 | - |  |  |  |  |  |  |  |  |  |  |  |  |  |  |  |
| ORF091 | - | 78899 | 77550 | 1350 | 449 | 95% | 94% | 95% | 30% | 30% | 30% | Primase/helicase (T4 gp41-like) | Phage ATP-dependent primase-helicase [*Campylobacter* phage CP220] | 0.0 | 428/449 (95%) | CBJ93934.1 |
| ORF092 | - | 79834 | 79055 | 780 | 259 | 97% | 97% | 98% | - | - | - | GTP cyclohydrolase | GTP cyclohydrolase [*Campylobacter* phage vB_CcoM-IBB_35] | 0.0 | 253/259 (98%) | AEI88255.1 |
| ORF093 | - | 80275 | 79844 | 432 | 143 | 99% | 99% | 98% | - | - | - | Non-specific DNA-binding protein Dps / Iron-binding ferritin-like antioxidant protein / Ferroxidase | Hypothetical phage protein [*Campylobacter* phage CP220] | 4,00E-99 | 141/143 (99%) | CBJ93932.1 |
| ORF094 | - | 80887 | 80300 | 588 | 195 | 100% | 100% | 98% | - | - | - | Sliding clamp protein (T4 gp45-like) | Sliding clamp [*Campylobacter* phage CP220] | 5,00E-136 | 195/195 (100%) | CBJ93931.1 |
| ORF095 | - | 81300 | 80989 | 312 | 103 | 96% | 98% | 97% | - | - | - | Unknown | Hypothetical phage protein [*Campylobacter* phage CPt10] | 1,00E-64 | 101/103 (98%) | CBJ94328.1 |
| ORF096 | - | 82095 | 81436 | 660 | 219 | 97% | 97% | 94% | - | 28% | 28% | Unknown | Hypothetical phage protein [*Campylobacter* phage CP220] | 9,00E-124 | 213/219 (97%) | CBJ93929.1 |
| ORF097 | - | 82405 | 82085 | 321 | 106 | 99% | 100% | 99% | - | - | - | Unknown | Hypothetical phage protein [*Campylobacter* phage CPt10] | 8,00E-68 | 106/106 (100%) | CBJ94326.1 |
| ORF098 | - | 83175 | 82468 | 708 | 235 | 99% | 100% | 99% | 31% | 30% | 30% | Neck protein (T4 gp13-like) | Phage neck protein [*Campylobacter* phage CPt10] | 2,00E-172 | 235/235 (100%) | CBJ94325.1 |
| ORF099 | - | 83774 | 83175 | 600 | 199 | 93% | 95% | 97% | - | - | - | Unknown | Hypothetical protein [*Campylobacter* phage vB_CcoM-IBB_35] | 8,00E-127 | 193/199 (97%) | AEI88262.1 |
| ORF100 | - | 84585 | 83806 | 780 | 259 | 98% | 99% | 98% | - | - | - | Unknown | Hypothetical phage protein [*Campylobacter* phage CPt10] | 4,00E-172 | 241/243 (99%) | CBJ94323.1 |
| ORF101 | - | 84821 | 84585 | 237 | 78 | - | 77% | 83% | - | - | - | Membrane protein | Hypothetical protein [*Campylobacter* phage vB_CcoM-IBB_35] | 3,00E-26 | 72/87 (83%) | AEI88265.1 |
| ORF102 | - | 85720 | 84815 | 906 | 301 | 98% | 97% | 97% | - | - | - | DNA polymerase (T4 gp43-like) | Phage DNA polymerase [*Campylobacter* phage CPt10] | 1,00E-174 | 255/263 (97%) | CBJ94321.1 |
| ORF103 | - | 86718 | 85717 | 1002 | 333 | - | - | - | - | - | - | Unknown | Hypothetical protein MB2181_05340 [*Methylophilales bacterium* HTCC2181] | 4,00E-05 | 33/83 (40%) | ZP_01552417.1 |
| ORF104 | - | 88918 | 86711 | 2208 | 735 | 93% | 92% | 92% | - | - | - | DNA polymerase | Possible phage DNA polymerase [*Campylobacter* phage CP220] | 0.0 | 625/674 (93%) | CBJ93924.1 |
| ORF105 | - | 89697 | 89038 | 660 | 219 | 93% | 92% | 92% | - | - | - | ExsB-like transcriptional regulator | Hypothetical phage protein [*Campylobacter* phage CP220] | 2,00E-148 | 203/219 (93%) | CBJ93923.1 |
| ORF106 | + | 90326 | 90649 | 324 | 107 | 94% | 97% | - | - | - | - | Membrane protein | Hypothetical phage membrane protein [*Campylobacter* phage CPt10] | 2,00E-67 | 104/107 (97%) | CBJ94316.1 |
| ORF107 | + | 90659 | 90937 | 279 | 92 | 91% | 91% | - | - | - | - | Lipoprotein | Hypothetical phage lipoprotein [*Campylobacter* phage CPt10] | 3,00E-55 | 84/92 (91%) | CBJ94317.1 |
| ORF108 | + | 90934 | 91662 | 729 | 242 | 91% | 89% | - | - | - | - | Unknown | Hypothetical phage protein [*Campylobacter* phage CP220] | 2,00E-148 | 226/247 (91%) | CBJ93921.1 |
| ORF109 | + | 91662 | 91886 | 225 | 74 | 94% | 94% | - | - | - | - | Unknown | Hypothetical phage protein [*Campylobacter* phage CP220] | 4,00E-35 | 62/66 (94%) | CBJ93922.1 |
| ORF110 | + | 92419 | 93201 | 783 | 260 | 93% | 95% | 95% | - | - | - | Membrane protein | Hypothetical phage membrane protein [*Campylobacter* phage CPt10] | 6,00E-178 | 248/260 (95%) | CBJ94315.1 |
| ORF111 | - | 95285 | 93198 | 2088 | 695 | 90% | 91% | 90% | - | - | - | Unknown | Hypothetical protein [*Campylobacter* phage CPt10] | 0.0 | 639/699 (91%) | CBJ94314.1 |
| ORF112 | - | 95486 | 95325 | 162 | 53 | 91% | 89% | 89% | - | - | - | Unknown | Hypothetical phage protein [*Campylobacter* phage CP220] | 8,00E-25 | 48/53 (91%) | CBJ93916.1 |
| ORF113 | - | 96380 | 95661 | 720 | 239 | 93% | 94% | 81% | - | - | - | Unknown | Hypothetical phage protein [*Campylobacter* phage CPt10] | 8,00E-166 | 224/239 (94%) | CBJ94312.1 |
| ORF114 | + | 96544 | 98310 | 1767 | 588 | 95% | 95% | 86% | - | - | - | Unknown | Hypothetical phage protein [*Campylobacter* phage CPt10] | 0.0 | 556/583 (95%) | CBJ94310.1 |
| ORF115 | - | 99956 | 98442 | 1515 | 504 | 96% | 95% | 91% | - | - | - | Tail fiber protein | Hypothetical phage protein [*Campylobacter* phage CP220] | 0.0 | 482/504 (96%) | CBJ93912.1 |
| ORF116 | - | 100463 | 100035 | 429 | 142 | 89% | 89% | 88% | - | - | - | Unknown | Hypothetical phage protein [*Campylobacter* phage CPt10] | 1,00E-84 | 126/142 (89%) | CBJ94307.1 |
| ORF117 | - | 103028 | 100557 | 2472 | 823 | 92% | 95% | 89% | - | - | - | Unknown | Hypothetical phage protein [Campylobacter phage CPt10] | 0.0 | 778/823 (95%) | CBJ94306.1 |
| ORF118 | - | 104244 | 103084 | 1161 | 386 | 97% | 97% | 98% | - | - | - | Unknown | Hypothetical protein [*Campylobacter* phage vB_CcoM-IBB_35] | 0.0 | 377/386 (98%) | AEF56792.1 |
| ORF119 | - | 105243 | 104284 | 960 | 319 | 99% | 98% | 99% | - | - | - | Radical SAM domain-containing protein | Hypothetical phage protein (Radical SAM family) [*Campylobacter* phage 220] | 0.0 | 316/319 (99%) | CBJ93908.1 |
| ORF120 | - | 105937 | 105266 | 672 | 223 | 95% | 95% | 95% | - | - | - | Unknown | Hypothetical protein [*Campylobacter* phage vB_CcoM-IBB_35] | 4,00E-148 | 208/218 (95%) | AEF56790.1 |
| ORF121 | - | 106170 | 105937 | 234 | 77 | 92% | 95% | 94% | - | - | - | Unknown | Hypothetical protein [*Campylobacter* phage vB_CcoM-IBB_35] | 7,00E-45 | 72/77 (94%) | AEF56789.1 |
| ORF122 | - | 106634 | 106185 | 450 | 149 | 96% | 97% | 95% | - | - | - | Unknown | Hypothetical phage protein [*Campylobacter* phage CP220] | 2,00E-100 | 143/149 (96%) | CBJ93905.1 |
| ORF123 | - | 107110 | 106718 | 394 | 131 | 69% | - | 90% | - | - | - | Membrane protein | Hypothetical protein [*Campylobacter* phage vB_CcoM-IBB_35] | 7,00E-34 | 117/130 (90%) | AEF56787.1 |
| ORF124 | - | 107448 | 107152 | 297 | 98 | 98% | 98% | 100% | - | - | - | Unknown | Hypothetical protein [*Campylobacter* phage vB_CcoM-IBB_35] | 3,00E-63 | 98/98 (100%) | AEF56786.1 |
| ORF125 | - | 108049 | 107657 | 393 | 130 | 96% | 96% | 95% | - | - | - | Unknown | Hypothetical phage protein [*Campylobacter* phage CP220] | 9,00E-58 | 74/77 (96%) | CBJ93902.1 |
| ORF126 | - | 108540 | 108082 | 459 | 152 | 37% | 43% | 35% | - | - | - | Unknown | Hypothetical phage protein [*Campylobacter* phage CP220] | 3,00E-18 | 48/129 (37%) | CBJ93900.1 |
| ORF127 | - | 109014 | 108562 | 453 | 150 | - | - | - | - | - | - | Unknown | - | - | - | - |
| ORF128 | - | 109507 | 109244 | 264 | 87 | 46% | - | - | - | - | - | Radical SAM domain-containing protein | Radical SAM domain protein [*Desulfobacter postgatei* 2ac9] | 2,00E-05 | 21/57 (37%) | ZP_09098248.1 |
| ORF129 | - | 109898 | 109500 | 399 | 132 | - | - | - | - | - | - | Unknown | - | - | - | - |
| ORF130 | - | 110272 | 110072 | 201 | 66 | - | - | - | - | - | - | Unknown | AstB/chuR-related protein [*Thermosipho africanus* TCF52B] | 1,00E-07 | 30/67 (45%) | YP_002334168.1 |
| ORF131 | - | 111156 | 110269 | 888 | 295 | - | 24% | - | - | - | - | Radical SAM domain-containing protein | Radical SAM domain protein [*Vibrio furnissii* NCTC 11218] | 7,00E-07 | 76/315 (24%) | ADT88960.1 |
| ORF132 | - | 111876 | 111163 | 714 | 237 | - | 37% | - | - | - | - | radical SAM protein? | 4Fe-4S single cluster domain protein [*Bacteriovorax* sp. BAL6_X] | 2,00E-04 | 39/117 (33%) | WP_021266015.1 |
| ORF133 | - | 112066 | 111920 | 147 | 48 | - | - | - | - | - | - | Unknown | - | - | - | - |
| ORF134 | - | 113004 | 112069 | 936 | 311 | - | 96% | 91% | - | - | - | Radical SAM domain-containing protein | Hypothetical phage protein (Radical SAM family) [*Campylobacter* phage CPt10] | 6,00E-172 | 296/309 (96%) | CBJ94288.1 |
| ORF135 | - | 113574 | 113017 | 558 | 185 | - | - | - | - | - | - | Unknown | - | - | - | - |
| ORF136 | - | 113695 | 113516 | 180 | 59 | 96% | - | - | - | - | - | Unknown | Hypothetical phage protein [*Campylobacter* phage CP220] | 4,00E-05 | 22/23 (96%) | CBJ93888.1 |
| ORF137 | + | 113961 | 113728 | 234 | 77 | 96% | 98% | 71% | - | - | - | Unknown | Hypothetical protein [*Campylobacter* phage CP220] | 3,00E-27 | 69/72 (96%) | CBJ93888.1 |
| ORF138 | - | 114247 | 114020 | 228 | 75 | - | - | 42% | - | - | - | Unknown | Hypothetical phage protein [*Campylobacter* phage vB_CcoM-IBB_35] | 6,00E-03 | 28/66 (42%) | AEF56776.1 |
| ORF139 | - | 114524 | 114399 | 126 | 41 | 100% | 98% | - | - | - | - | Unknown | Hypothetical phage protein [*Campylobacter* phage CP220] | 3,00E-15 | 41/41 (100%) | CBJ93887.1 |
| ORF140 | - | 115532 | 114744 | 789 | 262 | 95% | 98% | 95% | - | - | - | Unknown | Hypothetical phage protein [*Campylobacter* phage CPt10] | 4,00E-180 | 249/253 (98%) | CBJ94285.1 |
| ORF141 | - | 115833 | 115582 | 252 | 83 | 89% | 99% | 84% | - | - | - | Unknown | Hypothetical phage protein [*Campylobacter* phage CPt10] | 3,00E-39 | 82/83 (99%) | CBJ94284.1 |
| ORF142 | - | 116151 | 115858 | 294 | 97 | 95% | 99% | 96% | 48% | 48% | 48% | Phospholipase | Hypothetical phage protein [*Campylobacter* phage CPt10] | 2,00E-59 | 96/97 (99%) | CBJ94283.1 |
| ORF143 | - | 116576 | 116151 | 426 | 141 | 85% | 82% | 87% | - | - | - | Unknown | Hypothetical phage membrane protein [*Campylobacter* phage CP220] | 7,00E-71 | 122/143 (85%) | CBJ93883.1 |
| ORF144 | - | 117485 | 116601 | 885 | 294 | 99% | 98% | 99% | 23% | 21% | 21% | Baseplate hub subunit and tail lysozyme (T4 gp5-like) | Putative Rhs element Vgr family protein [*Campylobacter* phage vB_CcoM-IBB_35] | 0.0 | 290/294 (99%) | AEF56769.1 |
| ORF145 | - | 118177 | 117599 | 579 | 192 | 95% | 95% | 95% | - | - | - | Transketolase? | Hypothetical phage protein [*Campylobacter* phage CPt10] | 1,00E-128 | 181/190 (95%) | CBJ94280.1 |
| ORF146 | - | 118582 | 118217 | 366 | 121 | 95% | 91% | 79% | 42% | 38% | 41% | Unknown | Hypothetical phage protein [*Campylobacter* phage CP220] | 2,00E-71 | 115/121 (95%) | CBJ93880.1 |
| ORF147 | - | 119152 | 118616 | 537 | 178 | - | 99% | 97% | - | - | - | Unknown | Hypothetical phage protein [*Campylobacter* phage CPt10] | 3,00E-97 | 176/178 (99%) | CBJ94278.1 |
| ORF148 | - | 119884 | 119216 | 669 | 222 | 98% | 99% | 96% | - | - | - | Unknown | Hypothetical phage protein [*Campylobacter* phage CPt10] | 1,00E-151 | 220/222 (99%) | CBJ94277.1 |
| ORF149 | - | 120728 | 120123 | 606 | 201 | 100% | 44% | 44% | - | - | - | 6-pyruvoyl-tetrahydropterin synthase | Hypothetical phage protein [*Campylobacter* phage CP220] | 5,00E-147 | 201/201 (100%) | CBJ93878.1 |
| ORF150 | - | 121729 | 120743 | 987 | 328 | 99% | 99% | 100% | - | - | - | MreB-like ATPase involved in cell division | Hypothetical protein [*Campylobacter* phage vB_CcoM-IBB_35] | 0.0 | 328/328 (100%) | AEF56763.1 |
| ORF151 | - | 122267 | 121785 | 483 | 160 | 94% | 92% | 97% | - | - | - | Unknown | Hypothetical phage protein [*Campylobacter* phage CP220] | 4,00E-101 | 154/163(94%) | CBJ93874.1 |
| ORF152 | - | 122548 | 122264 | 285 | 94 | - | - | - | - | - | - | Unknown | - | - | - | - |
| ORF153 | - | 122747 | 122577 | 171 | 56 | 64% | 66% | 64% | 65% | - | 57% | Unknown | Hypothetical phage protein [*Campylobacter* phage CPt10] | 4,00E-17 | 37/56 (66%) | CBJ94273.1 |
| ORF154 | - | 123357 | 122791 | 567 | 188 | - | - | - | - | - | - | N6-adenosine-methyltransferase | Adenine-specific DNA methyltransferase [*Acaryochloris* sp. CCMEE 5410] | 5,00E-10 | 43/154 (28%) | ZP_09252796.1 |
| ORF155 | - | 124139 | 123399 | 741 | 246 | - | 97% | 92% | - | - | - | Unknown | Hypothetical phage protein [*Campylobacter* phage CPt10] | 6,00E-166 | 233/239 (97%) | CBJ94267.1 |
| ORF156 | - | 124627 | 124136 | 492 | 163 | - | 43% | 52% | 41% | 44% | 44% | Unknown, YopX family protein | Phage protein, partial [*Campylobacter coli* 86119] | 2,00E-48 | 91/159 (57%) | EIA58944.1 |
| ORF157 | - | 124978 | 124697 | 282 | 93 | - | 94% | 94% | - | - | - | Unknown | Hypothetical protein [*Campylobacter* phage vB_CcoM-IBB_35] | 1,00E-54 | 87/93 (94%) | AEF56754.1 |
| ORF158 | - | 125709 | 125200 | 510 | 169 | 79% | 86% | 85% | 68% | 67% | 73% | Exonuclease | Hypothetical phage protein [*Campylobacter* phage CPt10] | 3,00E-78 | 146/169 (86%) | CBJ94264.1 |
| ORF159 | - | 126102 | 125890 | 213 | 70 | - | 96% | - | - | - | - | Unknown | Hypothetical phage protein [*Campylobacter* phage CPt10] | 2,00E-29 | 55/57 (96%) | CBJ94263.1 |
| ORF160 | - | 126501 | 126286 | 216 | 71 | 93% | - | 96% | - | - | - | Phosphate starvation-induced protein | PhoH family precursor [*Campylobacter* phage vB_CcoM-IBB_35] | 2,00E-39 | 68/71 (96%) | AEF56752.1 |
| ORF161 | - | 126634 | 126506 | 129 | 42 | - | - | 100% | - | - | - | Phosphate starvation-induced protein | PhoH family precursor [*Campylobacter* phage vB_CcoM-IBB_35] | 7,00E-15 | 35/35 (100%) | AEF56752.1 |
| ORF162 | - | 127752 | 126631 | 1122 | 373 | - | 97% | - | - | - | - | Transposase, Tn7_Tnp_TnsA_N superfamily? | Hypothetical phage protein [*Campylobacter* phage CPt10] | 0.0 | 362/373 (97%) | CBJ94262.1 |
| ORF163 | - | 127870 | 127721 | 150 | 49 | - | - | 93% | - | - | - | Phosphate starvation-induced protein | PhoH family precursor [*Campylobacter* phage vB_CcoM-IBB_35] | 7,00E-08 | 25/27 (93%) | AEF56752.1 |
| ORF164 | - | 129147 | 127903 | 1245 | 414 | 97% | - | 98% | - | - | - | Phosphate starvation-induced protein | PhoH family precursor [*Campylobacter* phage vB_CcoM-IBB_35] | 0.0 | 405/412 (98%) | AEF56752.1 |
| ORF165 | + | 129363 | 129992 | 630 | 209 | - | - | - | - | - | - | Transposase, IS607 family | Resolvase domain [*Thermoanaerobacter ethanolicus* JW 200] | 9,00E-60 | 94/189 (50%) | ZP_08210846.1 |
| ORF166 | + | 130024 | 131268 | 1245 | 414 | 93% | 95% | 94% | - | - | - | Transposase, IS605 family | ISCaje1 transposase [*Campylobacter* phage CPt10] | 0.0 | 393/414 (95%) | CBJ94374.1 |
| ORF167 | - | 131810 | 131310 | 501 | 166 | 99% | 100% | 99% | 40% | 40% | 40% | DNA end protector protein (T4 gp2-like) | Hypothetical phage protein [*Campylobacter* phage CPt10] | 1,00E-114 | 166/166 (100%) | CBJ94260.1 |
| ORF168 | - | 132453 | 131863 | 591 | 196 | 99% | 99% | 99% | 37% | 37% | 37% | Tail tube monomer (T4 gp19-like) | Possible phage tail tube protein [*Campylobacter* phage CP220] | 3,00E-144 | 195/196 (99%) | CBJ93867.1 |
| ORF169 | - | 132987 | 132862 | 126 | 41 | - | - | - | - | - | - | Unknown | - | - | - | - |
| ORF170 | - | 133676 | 133134 | 543 | 180 | 92% | 92% | 93% | - | - | - | Transcriptional regulator NadR | Hypothetical protein [*Campylobacter* phage vB_CcoM-IBB_35] | 1,00E-116 | 168/180 (93%) | AEF56749.1 |
| ORF171 | - | 134494 | 133718 | 777 | 258 | 96% | 96% | 96% | - | - | - | Unknown | Hypothetical phage protein [*Campylobacter* phage CPt10] | 0.0 | 248/258 (96%) | CBJ94257.1 |
| ORF172 | - | 135625 | 134603 | 1023 | 340 | 96% | 95% | 96% | 51% | 50% | 50% | Poly A polymerase | Hypothetical protein [*Campylobacter* phage vB_CcoM-IBB_35] | 0.0 | 323/338 (96%) | AEF56747.1 |
| ORF173 | - | 136869 | 135670 | 1200 | 399 | 50% | 50% | 97% | 41% | 43% | 41% | Homing endonuclease | Hef [*Campylobacter* phage vB_CcoM-IBB_35] | 8,00E-178 | 268/275 (97%) | AEF56746.1 |
| ORF174 | - | 138003 | 136882 | 1122 | 373 | 27% | 27% | 82% | - | - | - | Homing endonuclease | Hef [*Campylobacter* phage vB_CcoM-IBB_35] | 1,00E-86 | 143/175 (82%) | AEF56746.1 |
| ORF175 | - | 138271 | 137987 | 285 | 94 | - | - | 88% | - | - | - | Homing endonuclease | Hef [*Campylobacter* phage vB_CcoM-IBB_35] | 7,00E-44 | 78/89 (88%) | AEF56746.1 |
| ORF176 | - | 138812 | 138375 | 438 | 145 | 96% | 97% | 96% | 53% | 54% | 54% | Tail sheath protein (T4 gp18-like) | Possible phage tail sheath protein [*Campylobacter* phage CPt10] | 3,00E-90 | 136/140 (97%) | CBJ94254.1 |
| ORF177 | - | 140313 | 139147 | 1167 | 388 | - | - | - | - | - | - | Unknown | Hypothetical protein [Enterobacteria phage Bp7] | 6,00E-08 | 32/79 (41%) | AEN93956.1 |
| ORF178 | - | 141744 | 140407 | 1338 | 445 | 98% | 98% | 97% | 39% | 40% | 40% | Tail sheat protein (T4 gp18-like) | Possible phage tail sheath protein [*Campylobacter* phage CP220] | 0.0 | 431/439 (98%) | CBJ93862.1 |
| ORF179 | - | 143667 | 142087 | 1581 | 526 | 67% | 67% | 67% | 39% | 39% | 39% | Tail sheat protein (T4 gp18-like) | Possible phage tail sheath protein [*Campylobacter* phage CP220] | 0.0 | 346/516 (67%) | CBJ93861.1 |
| ORF180 | - | 145088 | 143844 | 1245 | 414 | 97% | 97% | 97% | 42% | 41% | 41% | Major capsid protein (T4 gp23-like) | gp23 major capsid protein [*Campylobacter* phage vB_CcoM-IBB_35] | 0.0 | 403/414 (97%) | AEF56742.1 |
| ORF181 | - | 146003 | 145251 | 753 | 250 | 86% | 90% | 84% | - | - | - | Major prohead-scaffolding core protein (T4 gp22-like) | Possible prohead core scaffold protein [*Campylobacter* phage CPt10] | 7,00E-128 | 225/250 (90%) | CBJ94251.1 |
| ORF182 | - | 146430 | 146176 | 255 | 84 | 99% | 99% | 99% | - | - | - | Unknown | Hypothetical phage protein [*Campylobacter* phage CP220] | 3,00E-52 | 83/84 (99%) | CBJ93858.1 |
| ORF183 | - | 147194 | 146427 | 768 | 255 | 94% | 94% | 93% | 50% | 39% | 50% | Sigma factor for T4 late transcription (T4 gp55-like) | Hypothetical phage protein [*Campylobacter* phage CPt10] | 3,00E-174 | 240/255 (94%) | CBJ94249.1 |
| ORF184 | - | 147920 | 147261 | 660 | 219 | 96% | 96% | 97% | - | - | - | Unknown | Hypothetical protein [*Campylobacter* phage vB_CcoM-IBB_35] | 4,00E-143 | 213/219 (97%) | AEF56736.1 |
| ORF185 | - | 148267 | 148133 | 135 | 44 | - | - | - | - | - | - | Unknown | - | - | - | - |
| ORF186 | + | 149261 | 150142 | 882 | 293 | 85% | 82% | 86% | - | - | - | Unknown | Hypothetical phage protein [*Campylobacte*r phage CP220] | 1,00E-171 | 248/293 (85%) | CBJ93970.1 |
| ORF187 | + | 150210 | 151118 | 909 | 302 | 93% | 92% | 90% | - | - | - | NAD-dependent epimerase/dehydratase | Hypothetical phage protein [*Campylobacter* phage CPt10] | 0.0 | 279/302 (92%) | CBJ94369.1 |
| ORF188 | + | 151156 | 151728 | 573 | 190 | 95% | 95% | 93% | - | - | - | LmbE family protein | Hypothetical phage protein [*Campylobacter* phage CPt10] | 1,00E-129 | 181/190 (95%) | CBJ94370.1 |
| ORF189 | + | 151761 | 152306 | 546 | 181 | 99% | 99% | 99% | - | - | - | Polysaccharide deacetylase | Putative polysaccharide deacetylase [*Campylobacter* phage vB_CcoM-IBB_35] | 1,00E-128 | 180/181 (99%) | AEF56826.1 |
| ORF190 | + | 152373 | 153008 | 636 | 211 | 97% | 95% | 97% | - | - | - | FAD-dependent thymidylate synthase | Hypothetical phage protein [*Campylobacter* phage CP220] | 8,00E-129 | 204/211 (97%) | CBJ93974.1 |
| ORF191 | + | 153024 | 153251 | 228 | 75 | 69% | 68% | 73% | - | - | - | Unknown | Hypothetical phage protein [*Campylobacter* phage CP220] | 7,00E-19 | 51/74 (69%) | CBJ93975.1 |
| ORF192 | + | 153313 | 154422 | 1110 | 369 | 99% | 99% | 99% | - | - | - | Ribonucleotide reductase, small subunit | Ribonucleotide reductase, small subunit [*Campylobacter* phage CP220] | 0.0 | 368/369 (99%) | CBJ93977.1 |
| ORF193 | + | 154503 | 154910 | 408 | 135 | 93% | 97% | 95% | - | - | - | Unknown | Hypothetical phage protein [*Campylobacter* phage CPt10] | 2,00E-89 | 131/135 (97%) | CBJ94376.1 |
| ORF194 | - | 156127 | 154979 | 1149 | 382 | 99% | 99% | 99% | - | - | - | Unknown | Hypothetical phage protein [*Campylobacter* phage CPt10] | 0.0 | 378/382 (99%) | CBJ94377.1 |
| ORF195 | - | 156843 | 156127 | 717 | 238 | 99% | 99% | 98% | 33% | 32% | 32% | Head completion protein (T4 gp41-like) | Hypothetical protein [*Campylobacter* phage vB_CcoM-IBB_35] | 2,00E-167 | 233/238 (98%) | AEF56820.1 |
| ORF196 | + | 156890 | 158350 | 1461 | 486 | 89% | 91% | 71% | - | - | - | Tail fibre protein | Hypothetical phage protein [*Campylobacter* phage CP220] | 0.0 | 430/481 (89%) | CBJ93981.1 |
| ORF197 | + | 158304 | 162155 | 3852 | 1283 | 90% | 90% | 93% | 81% | 91% | 91% | Tail fibre protein | Hypothetical protein [*Campylobacter* phage vB_CcoM-IBB_35] | 0.0 | 1176/1270 (93%) | AEF56819.1 |
| ORF198 | + | 162327 | 162563 | 237 | 78 | - | - | - | - | - | - | Unknown | - | - | - | - |
| ORF199 | + | 162609 | 163967 | 1359 | 452 | 97% | 98% | 97% | 31% | 31% | 32% | Tail sheath stabilizer and completion protein (T4 gp15-like), partial | Possible phage tail sheath completion protein [*Campylobacter* phage CPt10] | 0.0 | 444/452 (98%) | CBJ94381.1 |
| ORF200 | + | 163976 | 164728 | 753 | 250 | 99% | 99% | 100% | 19% | 20% | 20% | Tail tube protein (T4 gp19-like) | gp19 tail tube protein [*Campylobacter* phage vB_CcoM-IBB_35] | 0.0 | 250/250 (100%) | AEF56817.1 |
| ORF201 | + | 164912 | 165418 | 507 | 168 | 99% | 98% | 98% | 31% | 31% | 31% | DNA topoisomerase II ATP-hydrolyzing DNA gyrase subunit B | Hypothetical phage protein [*Campylobacter* phage CP220] | 1,00E-92 | 167/168 (99%) | CBJ93985.1 |
| ORF202 | + | 165593 | 166510 | 918 | 305 | 93% | 94% | 94% | 38% | 38% | 38% | Single-stranded DNA binding protein (T4 gp32-like) | gp32 single-stranded DNA binding protein [*Campylobacter* phage vB_CcoM-IBB_35] | 0.0 | 291/310 (94%) | AEF56815.1 |
| ORF203 | + | 166551 | 166823 | 273 | 90 | 100% | 100% | 99% | - | - | - | Chaperonin Cpn10-like protein | Hypothetical phage protein [*Campylobacter* phage CP220] | 1,00E-56 | 90/90 (100%) | CBJ93987.1 |
| ORF204 | + | 166824 | 167273 | 450 | 149 | 90% | 91% | 81% | - | - | - | Transketolase | Hypothetical phage protein [*Campylobacter* phage CPt10] | 5,00E-95 | 139/153 (91%) | CBJ94386.1 |
| ORF205 | + | 167392 | 168996 | 1605 | 534 | 96% | 94% | 93% | - | - | - | N-acetylneuraminate pyruvate lyase | Hypothetical phage protein [*Campylobacter* phage CP220] | 0.0 | 512/534 (96%) | CBJ93989.1 |
| ORF206 | + | 169159 | 169881 | 723 | 240 | 97% | 97% | 97% | 48% | 47% | 47% | RnlA RNA ligase 1 and tail fiber attachment catalyst | RnlA RNA ligase 1 and tail fiber attachment catalyst [*Campylobacter* phage vB_CcoM-IBB_35] | 3,00E-156 | 224/232 (97%) | AEF56811.1 |
| ORF207 | + | 169919 | 170239 | 321 | 106 | 93% | 97% | 94% | - | - | - | RnlA RNA ligase 1 and tail fiber attachment catalyst | Phage RNA ligase [*Campylobacter* phage CPt10] | 5,00E-62 | 103/106 (97%) | CBJ94388.1 |
| ORF208 | + | 170393 | 170806 | 414 | 137 | 94% | 93% | 93% | - | - | - | Xanthine-guanine phosphoribosyltransferase | Hypothetical phage protein [*Campylobacter* phage CP220] | 1,00E-88 | 129/137 (94%) | CBJ93991.1 |
| ORF209 | + | 170816 | 171157 | 342 | 113 | 89% | 90% | 96% | - | - | - | Unknown | Hypothetical phage protein [*Campylobacter* phage CPt10] | 6,00E-68 | 102/113 (90%) | CBJ94390.1 |
| ORF210 | + | 171246 | 172415 | 1170 | 389 | 99% | 98% | 99% | - | - | - | Radical SAM domain-containing protein | Hypothetical phage protein (Radical SAM family) [*Campylobacter* phage CP220] | 0.0 | 388/389 (99%) | CBJ93993.1 |
| ORF211 | + | 172447 | 175995 | 3549 | 1182 | 90% | 97% | 94% | - | - | - | membrane protein? | Hypothetical phage membrane protein [*Campylobacter* phage CPt10] | 0.0 | 1138/1168 (97%) | CBJ94392 |
| ORF212 | + | 176023 | 176955 | 933 | 310 | 97% | 98% | 96% | - | - | - | Baseplate tail tube cap (T4 gp48-like) | Phage protein (possible baseplate tail tube cap) [*Campylobacter* phage CPt10] | 0.0 | 304/310 (98%) | CBJ94393.1 |
| ORF213 | + | 176964 | 177455 | 492 | 163 | 98% | 98% | 96% | - | - | - | Unknown | Hypothetical phage protein [*Campylobacter* phage CP220] | 3,00E-110 | 160/163 (98%) | CBJ93996.1 |
| ORF214 | + | 178424 | 178927 | 504 | 167 | 100% | 98% | 98% | 30% | 29% | 29% | Tail completion and sheath stabilizer protein (T4 gp3-like) | Phage tail completion and stabilizer protein [*Campylobacter* phage CP220] | 2,00E-118 | 167/167 (100%) | CBJ93997.1 |
| ORF215 | + | 178937 | 179356 | 420 | 139 | 98% | 98% | 99% | 31% | 32% | 32% | Unknown | Hypothetical phage protein [*Campylobacter* phage CP220] | 5,00E-90 | 136/139 (98%) | CBJ93998.1 |
| ORF216 | + | 179394 | 180749 | 1356 | 451 | 93% | 94% | 92% | - | - | - | Unknown | Hypothetical phage protein [*Campylobacter* phage CPt10] | 0.0 | 372/397 (94%) | CBJ94397.1 |
| ORF217 | + | 180827 | 181339 | 513 | 170 | 99% | 98% | 97% | 58% | 61% | 61% | Unknown | Hypothetical phage protein [*Campylobacter* phage CP220] | 1,00E-119 | 168/170 (99%) | CBJ94000.1 |
| ORF218 | + | 181348 | 181689 | 342 | 113 | 84% | 84% | 84% | - | - | - | Threonine dehydratase | Hypothetical phage protein [*Campylobacter* phage CP220] | 2,00E-50 | 81/97 (84%) | CBJ94001.1 |
| ORF219 | + | 181718 | 182473 | 756 | 251 | 91% | 90% | 99% | 99% | 55% | 55% | Prohead core scaffold and protease (T4 gp21-like) | Phage prohead core protein precursor [*Campylobacter* phage CP220] | 1,00E-148 | 227/249 (91%) | CBJ94002.1 |
| ORF220 | + | 182494 | 182700 | 207 | 68 | 85% | 82% | 84% | - | - | - | Unknown | Hypothetical phage protein [*Campylobacter* phage CPt10] | 7,00E-32 | 58/68 (85%) | CBJ94401.1 |

**Table S4. Primers used in this study**

| **Experiment** | **Primer** | **Nucleotide sequence (5‘-3‘)** | **Phage target region** |
| --- | --- | --- | --- |
| Repeat determination | CPGII_RR1-F | GTTTCAGATTCATTTGAAAATTTTATAAGTACAAAAT | RR1 (group II phages) |
|  | CPGII_RR1-R | AATATATTAGAATCTAATAAAACATTTGTTTTATGTG | RR1 (group II phages) |
|  | CPGII_RR2-F | CCAAAGAATATAACATAGCCAGCAG | RR2 (group II phages) |
|  | CPGII_RR2-R | CTTCGGGAACTGCTTTAGAAC | RR2 (group II phages) |
|  | CPGII_RR3-F | CTAAGTCTACAACAATTTTATCATTTTTAAAAGATT | RR3 (group II phages) |
|  | CPGII_RR3-R | TTTTAGTTCAAGTTCGTTAGTATAATCGTGTTTCAT | RR3 (group II phages) |
|  | CPGII_RR4-F | CCAAATGATTTCTATAAAAAAGTCAAAGA | RR4 (group II phages) |
|  | CPGII_RR4-R | CCTAGAAAAGGTAAAAATATTTTATAATTACTAGA | RR4 (group II phages) |
|  | CPGII_RR?_artifact-F | AAAAAGAATCTTAAAGCTATTCAAAAAGAATCTAAGA | RR?-artifact (CP21) |
|  | CPGII_RR?_artifact-R | TTATATTTTTTAAGAACTTCTAATTTATAATTAGAAC | RR?-artifact (CP21) |
| Contig structure determination | IBB_35_C3-F | CATTGTTTTTAACCTTTGGTTTATTTTATTAATAG | IBB_35 C3 – C4 |
|  | CPGII_RR1-R | AATATATTAGAATCTAATAAAACATTTGTTTTATGTG | IBB_35 C3 – C4 |
|  | IBB_35 C4-F | CTACCTGCAAAAAAGTCTAGGATAATATCATTCG | IBB_35 C4 – C5 |
|  | IBB_35 C5-R | CGGGAGTTCAAAATTTTTTAATCTCTTCTGCAG | IBB_35 C4 – C5 |
|  | IBB_35 C5-R | GGCTTAAAACTGTATCTTTCATATAAATCCTTTTGATTTG | IBB_35 C5 – C1 |
|  | CPGII_RR2-F | CCAAAGAATATAACATAGCCAGCAG | IBB_35 C5 – C1 |
|  | CPGII_RR4-F | CCAAATGATTTCTATAAAAAAGTCAAAGA | IBB_35 C2 – C3 |
|  | CPGII_RR4-R | CCTAGAAAAGGTAAAAATATTTTATAATTACTAGA | IBB_35 C2 – C3 |
|  | CPGII_RR3-R | TTTTAGTTCAAGTTCGTTAGTATAATCGTGTTTCAT | IBB_35 C1 – C2 |
|  | CPGII_RR1-F | GTTTCAGATTCATTTGAAAATTTTATAAGTACAAAAT | IBB_35 C1 – C2 |
| tRNA typing | CP21_tRNA-F | ACCACAACGATCATATTTACCG | tRNAs CP21-like |
|  | CP21_tRNA-R | TTATAATTTTCGGGGTGTAGCG | tRNAs CP21-like |
|  | CP220_tRNA-F | CTTCAGTGTTAGTTCCTGGCTC | tRNAs CP220-like |
|  | CP220_tRNA-R | AAGCTCGTCTAGTAAAGATTCG | tRNAs CP220-like |

**Table S5. Nucleotide differences within the group II tail tube genes (658 nt analysed).**

| **Position (nt)** | **CP21 subgroup** | | | **CP220 subgroup** | | | | | |
| --- | --- | --- | --- | --- | --- | --- | --- | --- | --- |
|  | **CP21** | CP83 | CP7 | **CP220** | CPt10 | IBB_35 | CP84 | CP75 | CP68 |
| 053 | A | A | A | G | A | A | A | A | A |
| 065 | C | C | C | T | T | T | T | T | T |
| 071 | A | A | A | T | T | T | T | T | T |
| 080 | A | A | A | A | G | A | G | G | G |
| 089 | T | T | T | C | C | C | C | C | C |
| 102 | G | G | G | A | A | A | A | A | A |
| 104 | A | A | A | C | C | C | C | C | C |
| 125 | T | T | T | T | T | C | T | T | T |
| 177 | A | A | A | C | C | C | C | C | C |
| 179 | A | A | A | T | T | T | T | T | T |
| 185 | A | A | A | G | G | G | G | G | G |
| 188 | C | C | C | T | T | T | T | T | T |
| 197 | C | C | C | T | T | T | T | T | T |
| 203 | A | A | A | A | C | G | C | C | C |
| 206 | A | A | A | A | T | A | T | T | T |
| 228 | A | A | A | T | T | A | T | T | T |
| 257 | C | C | C | T | T | T | T | T | T |
| 278 | A | A | A | G | G | A | G | G | G |
| 296 | T | T | T | T | T | C | T | T | T |
| 302 | T | T | T | A | G | T | G | G | G |
| 305 | C | C | C | T | T | C | T | T | T |
| 350 | A | A | A | C | C | C | C | C | C |
| 365 | C | C | C | T | T | T | T | T | T |
| 384 | T | T | T | T | T | A | T | T | T |
| 401 | G | G | G | A | A | A | A | A | A |
| 434 | A | A | A | G | G | A | G | G | G |
| 455 | C | C | C | T | T | T | T | T | T |
| 503 | G | G | G | C | C | C | C | C | C |
| 528 | G | G | G | A | A | A | A | A | A |
| 533 | G | G | G | A | A | A | A | A | A |
| 566 | C | C | C | T | C | C | C | C | C |
| 595 | G | G | G | A | A | A | A | A | A |
| 623 | T | T | T | A | A | A | A | A | A |
| 638 | T | T | T | C | C | C | C | C | C |
| 641 | T | T | T | C | C | C | C | C | C |
| 642 | T | T | T | C | C | C | C | C | C |
| 644 | G | G | G | A | A | A | A | A | A |
| 645 | A | A | A | G | G | G | G | G | G |
| 653 | C | C | C | T | T | T | T | T | T |

**Figure S1. Additional repeat region on the genomes of CP21, CP220 and CPt10.** Dot plot analyses of the additional repeat region of CP21 (A), CP220 (B) and CPt10 (C).


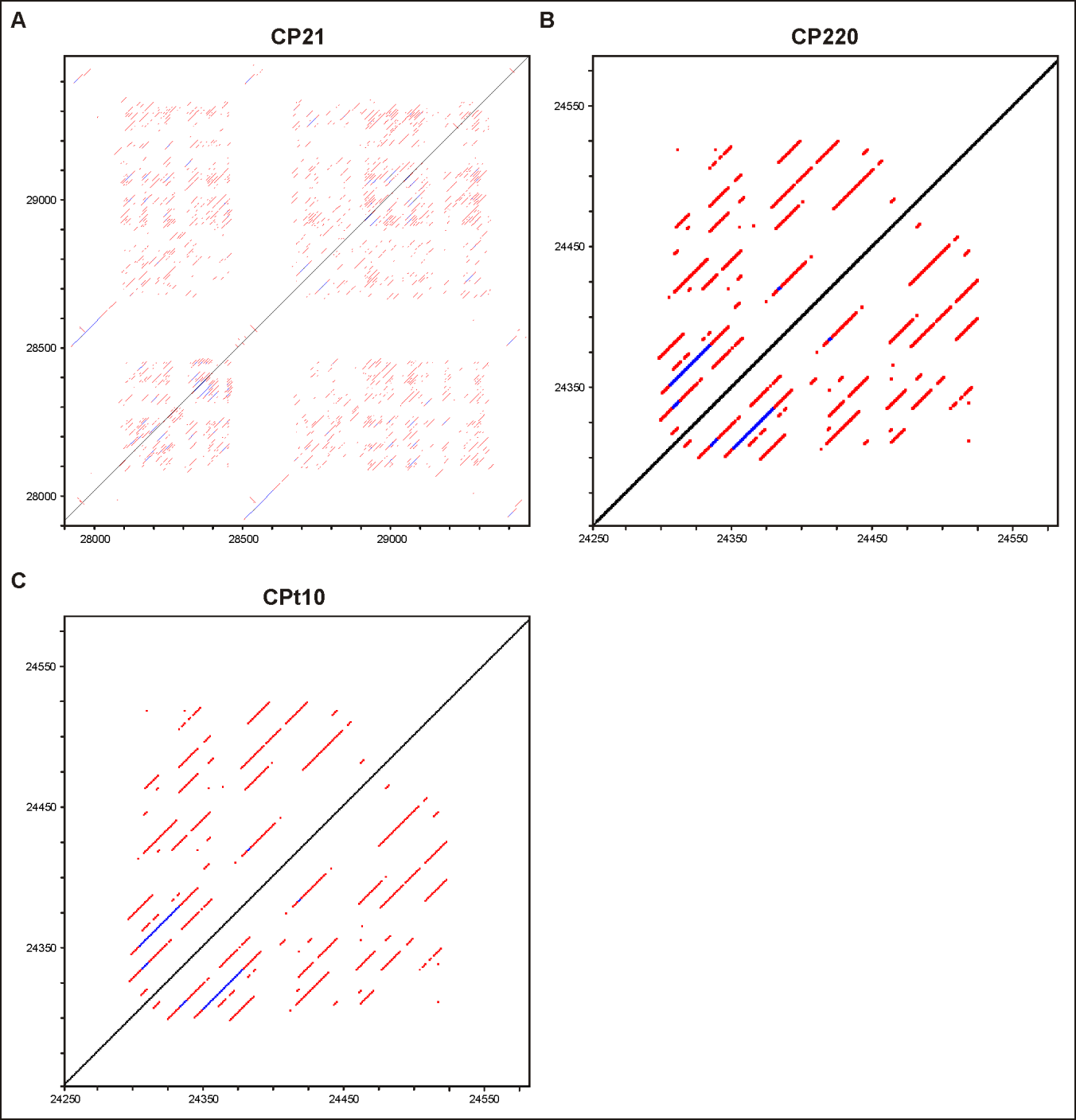

Supplement: Additional file 1: Table S1. — Strain specificity of Campylobacter phages. Table S2. CP21 DNA regions that are absent or relocated in the other group II phages. Table S3. CP21 ORF analysis. Table S4. Primers used in this study. Table S5. Nucleotide differences within the group II tail tube genes. Figure S1. Additional repeat region on the genomes of CP21, CP220 and CPt10. Dot plot analyses of the additional repeat region of CP21 (A), CP220 (B) and CPt10 (C). (DOCX 288 kb) [file 12864_2015_1837_MOESM1_ESM.docx]
